# Expression of Constitutively Active CDK1 Stabilizes APC-Cdh1 Substrates and Potentiates Premature Spindle Assembly and Checkpoint Function in G1 Cells

**DOI:** 10.1371/journal.pone.0033835

**Published:** 2012-03-29

**Authors:** Yan Ma, Xi Yuan, William R. Wyatt, Joseph R. Pomerening

**Affiliations:** 1 Department of Biology, Indiana University, Bloomington, Indiana, United States of America; 2 Department of Statistics, Indiana University, Bloomington, Indiana, United States of America; Duke University Medical Center, United States of America

## Abstract

Mitotic progression in eukaryotic cells depends upon the activation of cyclin-dependent kinase 1 (CDK1), followed by its inactivation through the anaphase-promoting complex (APC)/cyclosome-mediated degradation of M-phase cyclins. Previous work revealed that expression of a constitutively active CDK1 (CDK1AF) in HeLa cells permitted their division, but yielded G1 daughter cells that underwent premature S-phase and early mitotic events. While CDK1AF was found to impede the sustained activity of APC-Cdh1, it was unknown if this defect improperly stabilized mitotic substrates and contributed to the occurrence of these premature M phases. Here, we show that CDK1AF expression in HeLa cells improperly stabilized APC-Cdh1 substrates in G1-phase daughter cells, including mitotic kinases and the APC adaptor, Cdc20. Division of CDK1AF-expressing cells produced G1 daughters with an accelerated S-phase onset, interrupted by the formation of premature bipolar spindles capable of spindle assembly checkpoint function. Further characterization of these phenotypes induced by CDK1AF expression revealed that this early spindle formation depended upon premature CDK1 and Aurora B activities, and their inhibition induced rapid spindle disassembly. Following its normal M-phase degradation, we found that the absence of Wee1 in these prematurely cycling daughter cells permitted the endogenous CDK1 to contribute to these premature mitotic events, since expression of a non-degradable Wee1 reduced the number of cells that exhibited premature cyclin B1oscillations. Lastly, we discovered that Cdh1-ablated cells could not be forced into a premature M phase, despite cyclin B1 overexpression and proteasome inhibition. Together, these results demonstrate that expression of constitutively active CDK1AF hampers the destruction of critical APC-Cdh1 targets, and that this type of condition could prevent newly divided cells from properly maintaining a prolonged interphase state. We propose that this more subtle type of defect in activity of the APC-driven negative-feedback loop may have implications for triggering genome instability and tumorigenesis.

## Introduction

Proliferation of eukaryotic cells depends upon reiterative cycles of synthesis, activation, and inactivation/degradation of suites of regulators that direct growth, genome replication, and cell division. The family of enzymes that govern the transitions into and passage through these phases are the cyclin-dependent kinases (CDKs) – proteins activated by the binding of their regulatory subunit, cyclin. CDKs are classified according to the periods of the cell cycle they exert their catalytic control, with CDK1 stimulating M-phase initiation and progression [1]. The inactivating counterpart to CDK1 is the anaphase-promoting complex (APC), an E3 ubiquitin ligase that targets cyclins and other proteins for proteasome-mediated degradation during late M and early G1 phase. This APC-mediated negative-feedback loop drives mitotic progression, exit, and the maintenance of G1 phase [2]. The first active complex, APC-Cdc20, functions during early mitosis, degrading substrates including cyclin and securin; in somatic cells, Cdh1 then replaces Cdc20 as CDK1 activity diminishes during late mitosis. Once Cdh1 is dephosphorylated it associates with APC, and APC-Cdh1 remains active until Cdh1 itself is degraded in late G1 phase [3], [4]. APC-Cdh1 ubiquitylates substrates that include Cdc20 and the spindle-associating kinases Polo-like kinase 1 (Plk1), Aurora A (AurA), and Aurora B (AurB), and degrading the latter two is important for spindle reorganization during anaphase [5], [6].

APC activation is coupled to the dynamics of CDK1 activation, and prior studies showed the importance of this relationship in early embryonic and somatic cell cycle control [7], [8]. Amplification of CDK1 activity is critical to ensure APC is stimulated properly, and this requires positive feedback: upon mitotic entry CDK1 inhibits its inhibitor, Wee1 (and Myt1 kinase) [9], [10], [11], [12]—generating a double-negative (positive) feedback loop—and initiates another positive-feedback loop by activating its stimulatory phosphatase, Cdc25 [9], [10], [11], [12]. CDK1 activation can be made more gradual by introducing into cells or cell extracts a mutant CDK1—CDK1(T14A,Y15F) (CDK1AF)—that resists phosphorylation by the Wee1/Myt1 kinases, and this can perturb cell cycle progression. Previous work revealed that adding CDK1AF to embryonic extracts caused insufficient APC-Cdc20 activation during mitosis, producing damped CDK1 oscillations and incomplete periods of DNA replication [7]. CDK1AF expression in HeLa cells did not accelerate mitotic entry from G2 phase [8], [13], [14], [15]. However, division of CDK1AF-expressing cells produced G1 daughters with apparent defects in APC-Cdh1 activation, causing the slowed destruction of a Cdh1-specific target: an RFP-tagged securin lacking its D-box [8], [16]. This partial APC-Cdh1 activation corresponded with the onset of premature DNA replication that was interrupted by mitotic behaviors including chromatin condensation and nuclear envelope breakdown. But which substrates were improperly stabilized in these daughter cells remained unknown, as well as whether or not these conditions would permit mitotic spindle formation despite incomplete DNA replication. Together—as a result of defective APC-Cdh1 activation—premature mitotic entry concurrent with S phase in a cell could be catastrophic, due to an increased potential for genomic instability.

Since CDK1AF-expression had been shown previously to impede sustained APC-Cdh1 activation in G1-phase cells, we used this approach to determine if mitotic regulators that were improperly present following cell division could contribute to premature mitotic behaviors—such as spindle formation—that could promote chromosomal instability. Indeed, CDK1AF expression caused bipolar spindle formation in G1 daughters that had prematurely initiated but not completed DNA replication, and this coincided with the presence and proper localization of three mitotic kinases that are APC-Cdh1 substrates; Cdc20 was stabilized as well. Bipolar spindles not only formed in a majority of these G1 cells, but also generated a functioning spindle assembly checkpoint (SAC) that corresponded with active AurB and CDK1. Flow cytometry and live-cell imaging validated that these phenotypes occurred in G1 cells due to activation of endogenous CDK1 as a result of the normal absence of Wee1, and simply not as an idiosyncrasy of CDK1AF expression. Experiments knocking down Cdh1 protein revealed an elevation of cyclin B1 levels, but premature mitosis did not occur. We then discovered that Cdh1-ablated cells are incapable of entering mitosis during G1 and S phases, despite ectopic cyclin B1 overexpression and proteasome inhibition. These experiments confirmed that Cdh1 knockdown yields cells with accelerated S-phase onset, but showed for the first time that they remain capable of preventing aberrant mitotic onset. This is in contrast to CDK1AF expression causing newly divided cells to retain mitotic regulators that are APC-Cdh1 targets, resulting in the onset of premature mitotic events including spindle assembly.

Altogether, these findings revealed that complete APC-Cdh1 activation is important to prevent newly divided HeLa cells from overriding G1 phase with premature S-phase onset and early mitotic spindle formation. Without it, critical substrates are not degraded at M-phase completion, including cyclin B, mitotic kinases, and its partner APC regulator, Cdc20. It is clear that APC-Cdh1 activity is not solely for driving mitotic exit, but that its maintained activity is essential to reset new G1 cells completely from their prior mitotic state. This prevents disruptions in the cycles of daughter cells and avoids the possibility of a premature mitosis without completed DNA synthesis. Our study is novel in its finding that stabilizing APC-Cdh1 substrates can increase the susceptibility of a cell to premature S- and M-phase onset, and through bipolar spindle formation and SAC activation, could increase the likelihood of aneuploidy and cellular transformation.

## Results

### Mitosis of Cells Expressing CDK1AF Generates G1 Daughters with Stabilized APC-Cdh1 Substrates and Premature Spindle Formation

Eukaryotic cells govern precisely their progress through the cell cycle, particularly during cell division when mitotic kinases are active, and upon mitotic exit when these kinases are inactivated by degradation 5,17,18. In conjunction with APC-Cdc20, APC-Cdh1 targets substrates for proteolysis as cells complete mitosis. Of these targets, AurA localizes to spindle poles [19] and participates in centrosome maturation and spindle assembly [20], [21]. AurB localizes to the inner centromere during prometaphase and metaphase [22] and is required for proper chromosome alignment [23]. Lastly, Plk1 localizes to spindle poles and kinetochores during prometaphase and metaphase [24] and is necessary for spindle assembly and chromosome segregation [25], [26].

**Figure 1:**
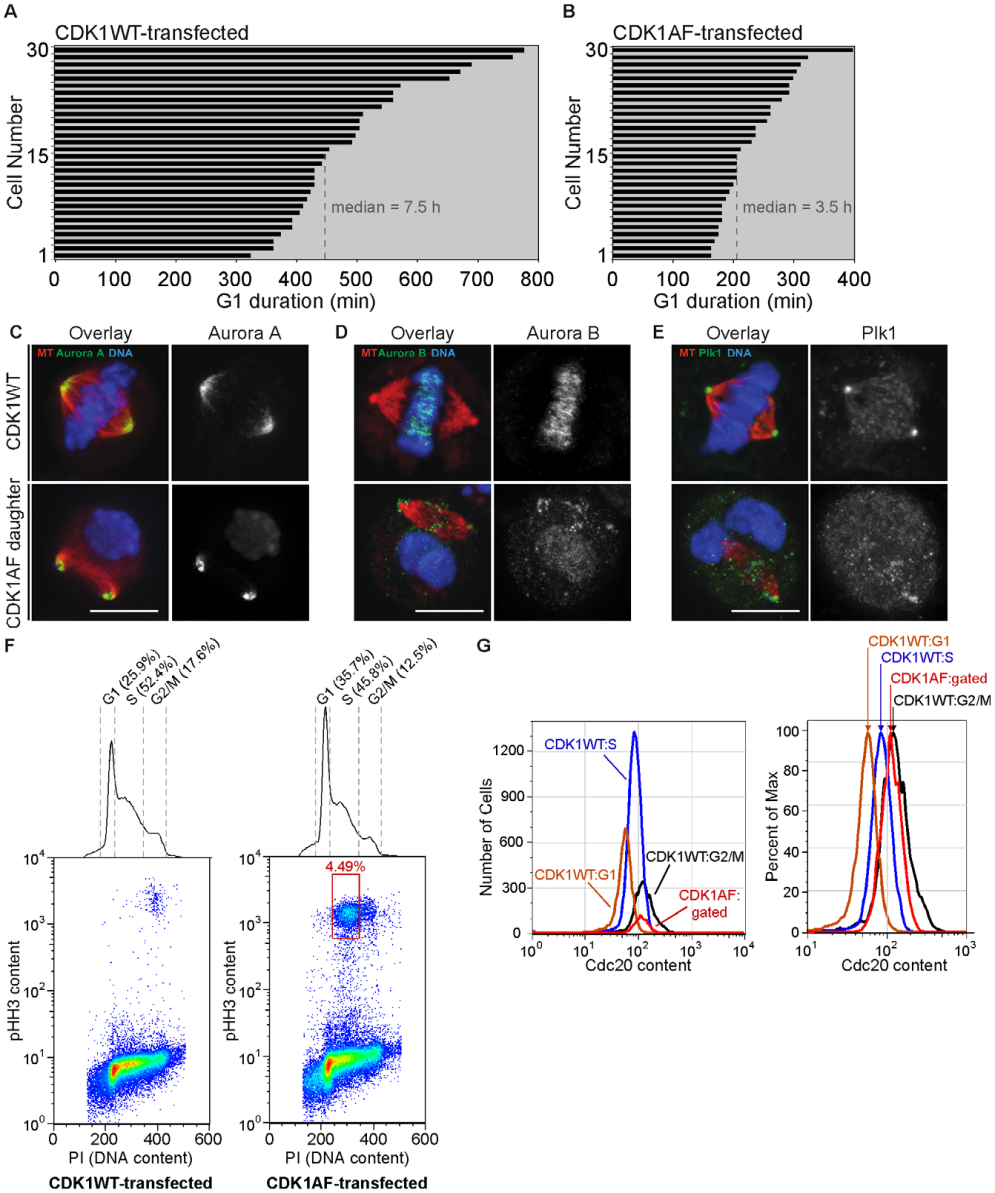
M-phase kinases are present and properly localized on mitotic spindles formed in G1 daughters with impaired APC-Cdh1 activity. (A) Histogram of G1-phase duration in daughters of CDK1WT-expressing HeLa cells. (B) Histogram of G1-phase duration in daughters of CDK1AF-expressing HeLa cells. (C) CDK1WT M-phase cell and M-phase-like G1 cell (CDK1AF daughter) probed for Aurora A, (D) Aurora B, and (E) Plk1; mCherry-α-tubulin (red) and kinases (green). (F) Pseudo-color scatter plots of DNA content vs. phospho-histone H3 (pHH3) content in 50000 CDK1WT-transfected (left) or CDK1AF-transfected (right) HeLa cells, with DNA content histogram. (G) Histogram of Cdc20 contents in cell cycle phases of CDK1WT-expressing cells and the low DNA-content/high pHH3-content CDK1AF-expressing cells (left); same histogram shown as percent of max (right).

Prior work revealed that division of cells expressing a constitutively active version of CDK1—CDK1AF—produced G1 daughters lacking sustained APC-Cdh1 activity [8]. We hypothesized that this defect might also be sufficient to facilitate premature mitotic events—including spindle formation—if mitotic regulators that are APC-Cdh1 targets are improperly stabilized. Using live-cell imaging and formation of nuclear PCNA puncta as an S-phase readout, we first measured the durations of G1 phase HeLa cells following transfection with CDK1WT or CDK1AF. CDK1WT-expression produced daughter cells with a median G1 duration of 7.5 h (449.5 min) (Figure 1A); the G1 duration of daughters of CDK1AF-expressing cells was 3.5 h (207.7 min) (Figure 1B). CDK1AF-transfected cells produced daughters that exhibited premature DNA condensation during these early S phases (Video S1), whereas CDK1WT-expressing cells cycled normally (Video S2).

CDK1WT- and CDK1AF-transfected populations were then fixed and scored for mitotic spindle formation, respectively, along with the presence and localizations of mitotic kinases that are known APC-Cdh1 substrates. CDK1WT-expressing cells showed the anticipated localizations on the mitotic spindle and chromosomes of AurA (Figure 1C, top), AurB (Figure 1D, top), and Plk1 (Figure 1E, top), as described above. Surprisingly, spindles formed in the G1 daughters of CDK1AF-expressing cells, and this corresponded with incomplete APC-Cdh1 function since mitotic kinases were inappropriately stabilized (Figure 1C–E, bottom). Additionally, their patterns of localization were nearly identical to control cells, with AurA at the spindle poles (Figure 1C, bottom), AurB at the inner centromeres—including those located on broken chromosomal arms found at the poles (Figure 1D, bottom)—and Plk1 at the spindle poles and kinetochores (Figure 1E, bottom). While AurB and Plk1 appear to be dispersed improperly—with some AurB proximal to the poles and some Plk1 appearing within the spindle—these kinases co-stained in the proper locations but also upon broken chromosome arms, presumably at centromeric regions where kinetochores are assembled (Figure S1).

In addition to the mitotic kinases targeted by APC-Cdh1, the APC co-activator Cdc20 is also ubiquitylated soon after anaphase onset [27]. In order to test if the observed premature spindle formation also coincided with the stabilization of Cdc20, we probed CDK1AF-expressing cells after division for the improper presence of this protein in G1 phase. Cells were also probed for phospho-Ser10-histone H3 (pHH3) content, a modification synonymous with M-phase chromosome condensation [28] (Figure 1F and G). CDK1WT transfection did not produce low DNA content/ pHH3-positive cells (Figure 1F, left), but CDK1AF transfection did (Figure 1F, right; see red boxed population), producing 4.49% of low DNA content/pHH3-positive cells (Figure 1G, left; red curve). Plotting the Cdc20 content of this premature mitotic population revealed higher levels of Cdc20 than in the G1- or S-phase cells of the CDK1WT-transfected population (Figure 1G, right; red curve), but slightly less than the G2/M cells in the CDK1WT-expressing population (Figure 1G, right; black curve). Altogether, these results demonstrate that division of cells expressing CDK1AF produced G1 cells that are capable of premature mitotic spindle formation, and this corresponds with the presence of stabilized APC-Cdh1 targets including mitotic kinases and Cdc20.

### Bipolar Spindles Can Be Maintained Irrespective of Chromatin Condensation State

**Figure 2:**
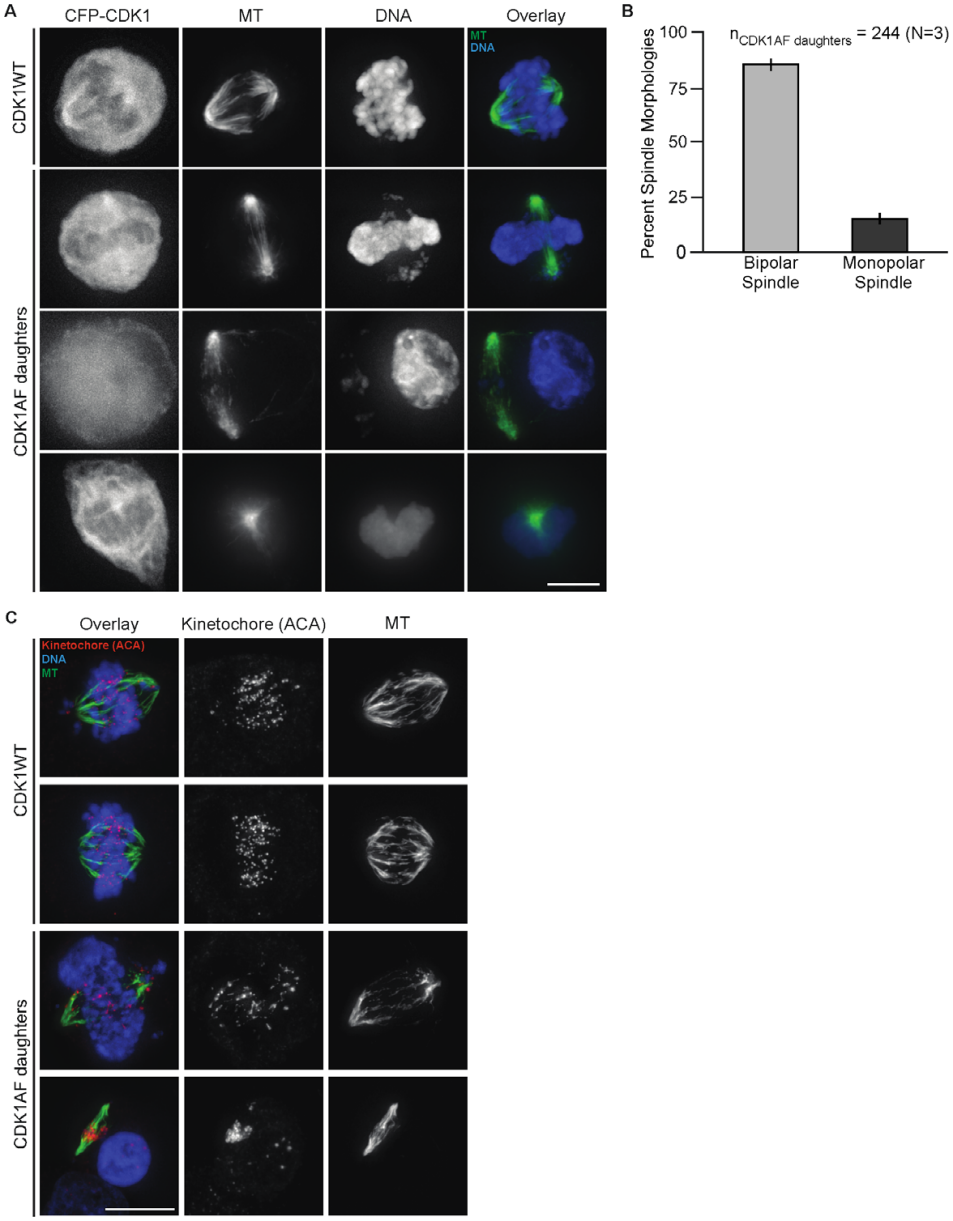
Bipolar spindle maintenance does not require completed DNA replication or condensation. (A) CDK1-CFP, microtubules (MT), DNA, and the overlay of MT and DNA in CDK1WT mitotic cells and daughters of CDK1AF expressers. Microtubules (green) and DNA (blue) are shown. (B) Histogram of spindle morphology observed in M-phase-like G1 cells (CDK1AF daughters), with percent bipolar (light gray) and monopolar spindles (dark gray). (C) Kinetochores (ACA; magenta), microtubules (MT), and the overlay of both of these plus DNA from cells in the same population described in (A). Scale bars = 10 µM.

Division of HeLa cells expressing constitutively active CDK1 caused the stabilization of APC-Cdh1 substrates—including mitotic kinases and Cdc20—in newly produced G1 cells. This permitted spindle formation, but whether or not these premature spindles were at all functional (e.g. might they generate a spindle checkpoint?) remained unclear. In order to characterize these spindles and the status of their accompanying chromosomes, live- and fixed-cell microscopy were performed on these daughter cells and CDK1WT-expressing controls. The latter underwent normal mitosis: chromosomes condensed and congressed to the spindle midzone as cells approached metaphase (Figure 2A, “CDK1WT”; Video S3). These processes in the daughter cells of CDK1AF-expressers were incoherent, and produced what appeared to be decondensed DNA at the spindle periphery, while some chromosomal fragments remained trapped within (Figure 2A, “CDK1AF”). Live-cell imaging revealed spindle structures were maintained in the presence of DNA (as scored by GFP-histone H2B) that was either condensed or in various stages of decondensation, with many cells containing detached peripheral nuclear bodies that were completely extruded from the spindle (Video S4). Surprisingly—despite entering a mitotic state a few hours after dividing and in what should be G1 phase—85% of daughters formed bipolar spindles, with the remaining 15% possessing monopolar spindles (Figure 2, A and B). While a majority of these G1 daughters formed bipolar spindles, they were narrower compared to those of control cells. This was most likely as a result of this M-phase state occurring prior to complete chromosome replication.

Division of CDK1AF-expressing cells produces G1 daughters that initiate DNA replication prematurely, and if these undergo mitosis prior to S phase completion, then many chromatids should remain unpaired. To test this idea, cells were stained with an anti-centromere antibody (ACA), and kinetochores were clearly paired and aligned along the metaphase plate in CDK1WT-expressing mitotic cells (Figure 2C, “CDK1WT”). However, division of CDK1AF-expressers produced G1 cells with precocious spindle formation and mostly unpaired kinetochores (Figure 2C, “CDK1AF daughters”). Individual kinetochores were located on chromosome fragments and maintained their spindle attachment while trapped at cell midzones (Figure S2), or alternatively, were spread throughout decondensed masses of chromatin at the spindle periphery. These results showed for the first time that bipolar spindle formation and kinetochore attachment could occur independent of DNA content or condensation state in G1-phase HeLa cells.

### G1-Phase Spindle Formation Requires Premature CDK1 and Aurora B Activities

**Figure 3:**
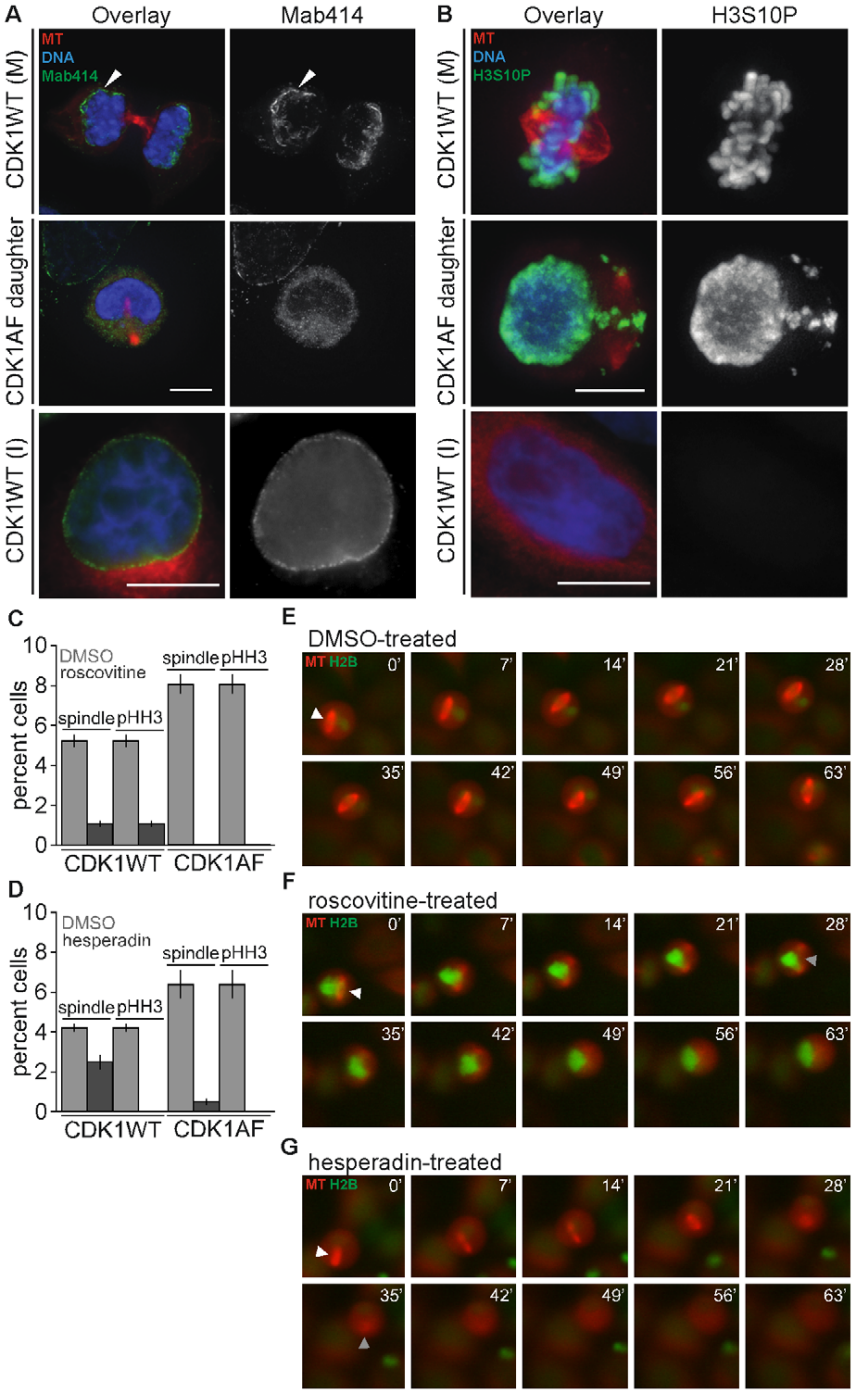
Premature spindle formation in G1 cells requires CDK1 and AurB activities. (A) Representative mitotic (M) and interphase (I) CDK1WT-expressing cells and a G1 cell produced by division of a CDK1AF-expresser (CDK1AF daughter), presenting microtubules (MT; red), DNA (blue), and nuclear pore complexes (Mab414; green). (B) Different cells from the population in (A), probed for pHH3 content (green). (C) Histogram of percent CDK1WT mitotic or CDK1AF M-phase-like G1 cells with spindles and/or pHH3 content after DMSO or roscovitine treatment. (D) Histogram of percent CDK1WT mitotic or CDK1AF M-phase-like G1 cells with spindles and/or pHH3 content after DMSO or hesperadin treatment. Unsynchronized HeLa cells stably expressing histone H2B-GFP (green) and mCherry-α-tubulin (red) were transfected with CFP-CDK1AF, then 24 h later were treated with DMSO, roscovitine, or hesperadin, and imaged live for 75 min (E–G). (E) M-phase-like G1 cell treated with DMSO. (F) M-phase-like G1 cell treated with roscovitine. (G) M-phase-like G1 cell treated with hesperadin. Scale bars in (A–C) = 10 µM. White arrowheads in (E–G) indicate prematurely formed spindle; gray arrowheads in (F–G) indicate last image of apparent spindle structure.

Cells expressing constitutively active CDK1 prior to mitosis produced daughters that entered S-phase prematurely, followed early mitotic onset and incoherent cell cycle behaviors: mitotic spindles co-existing with newly reformed interphase-like nuclear structures. But it was unclear which cell cycle state predominated in these cells, and unknown which kinase activities that enabled this spindle formation. Cells in early G1 normally form a nuclear envelope at the chromatin surface that contains lamins and nuclear pore complexes (NPCs) [29]. M-phase-like daughter cells stained for NPC proteins [30] revealed that they reformed nucleus-like bodies that had no pore complexes surrounding the chromosomes, indicating that their nuclei were not fully re-assembled (Figure 3A, “CDK1AF daughter”). All CDK1WT-expressing cells, however, had NPCs localized to the surface of the decondensing chromosomes soon after cytokinesis (Figure 3A, “CDK1WT (M)”), and later within the nuclear envelope (Figure 3A, “CDK1WT (I)”). While CDK1AF expression produced G1 daughters that re-formed nucleus-like bodies concomitant with mitotic spindles, this nuclear re-assembly remained incomplete relative to control cells.

G1 daughters produced by division of CDK1AF-expressing cells oscillated between interphase-like and M-phase-like states, but appeared to be incapable of resetting to an interphase state due to their possessing only partially assembled nuclear envelopes concurrent with mitotic spindles. Mitotic kinases were improperly stabilized in these G1 cells, and their normal localization suggested that along with CDK1 they could be active and promote early mitotic events, including premature spindle assembly. To determine whether these cells were in a physiological state representative of M phase, we probed these cells for pHH3 content. CDK1WT-expressing cells all stained positive for pHH3 in mitosis (Figure 3B, “CDK1WT (M)”), and none were pHH3-positive in interphase (Figure 3B, “CDK1WT (I)”). Spindle-bound chromosomal fragments as well as chromosomes localized to decondensed nucleus-like structures, however, were pHH3-positive in the G1 cells produced by division of CDK1AF-expressers (Figure 3B, “CDK1AF daughter”). Therefore, despite chromosome decondensation in these latter cells, the M-phase phosphorylation on Ser10 of HH3 was preserved.

With phosphorylation of HH3 perduring in these premature mitotic cells, it appeared that early activation of CDK1 along with a HH3 kinase might together drive the early spindle formation. We therefore tested whether inhibiting CDK1 or AurB—the latter a known HH3 kinase [31]—would promote spindle disassembly. First, HeLa cells stably expressing mCherry-α-tubulin were transfected with either CFP-CDK1WT or CFP-CDK1AF, and 24 h later were treated with either DMSO or roscovitine, a CDK1 inhibitor [32]. Cell populations were then surveyed for pHH3 and spindle content. Daughter cells produced in the CDK1AF-transfected population that were treated with DMSO stained positive for these M-phase markers, whereas roscovitine treatment eliminated spindles and pHH3 (Figure 3C). Both markers were strongly reduced in CDK1WT-expressing cells treated with roscovitine, with less than 1% still retaining spindles and pHH3 staining (Figure 3C) – this likely resulted from mitotic cells that had engaged the SAC but had insufficient time to release [33].

Premature CDK1 activity was found to be required for premature spindle formation in the G1 daughters of CDK1AF-expressing cells, and we next tested if AurB also contributed to this phenotype. AurB inhibition induces mitotic exit of cells arrested by the microtubule-stabilizing drug paclitaxel [23], so we hypothesized that treating the daughters of CDK1AF-expressing cells with hesperadin—an AurB inhibitor [34]—would reduce pHH3 levels and promote spindle disassembly. Similar to roscovitine treatment, hesperadin ablated HH3 phosphorylation and spindles in G1 cells produced by division of CDK1AF-expressers (Figure 3D). No pHH3 was detected in CDK1WT-expressing cells treated with hesperadin, and spindle formation was reduced relative to the DMSO control (Figure 3D).

Addition of either roscovitine or hesperadin decreased the percentage of G1 daughters that formed mitotic spindles following division of CDK1AF-expressing cells. To determine the time scale of this spindle disassembly, either DMSO or inhibitors were added just prior to imaging of HeLa cells expressing CDK1WT or CDK1AF. CDK1WT transfection and DMSO treatment caused no defects, whereas CDK1AF transfection followed by DMSO treatment produced daughters that formed premature and stabilized mitotic spindles (Figure 3E). Rapid spindle disassembly occurred when these cells were treated with either roscovitine (Figure 3F) or hesperadin (Figure 3G). Altogether, these results are novel in their revealing that two of the kinases critical for spindle formation during mitosis—CDK1 and AurB—which should both be inactive immediately following cell division, can indeed function in G1 cells. The improper activities of these kinases at this point of the cell cycle would require incomplete APC-Cdh1 function, with the premature reaccumulation of cyclin B1 needed to activate CDK1, as well as the stabilization of AurB during G1 phase.

### Knockdown of Mad2 Induces Rapid Spindle Disassembly in Precocious Cycling G1 Cells

Division of CDK1AF-expressers produced G1 cells that formed premature and unusually stable bipolar spindles. Live-cell imaging revealed a median duration of 250 min from spindle assembly to disassembly in these cells, relative to 35 minutes from metaphase to spindle disassembly at cytokinesis in CDK1WT-expressing cells (Figure 3 and Figure S3; Video S3 and S4). Either roscovitine or hesperadin treatment of these cells caused rapid spindle disassembly, implying that these spindles could be capable of some functionality. Together, these results led us to hypothesize that improper kinase activities during G1 phase might not only drive premature spindle assembly, but also contribute to spindle assembly checkpoint (SAC) function.

**Figure 4:**
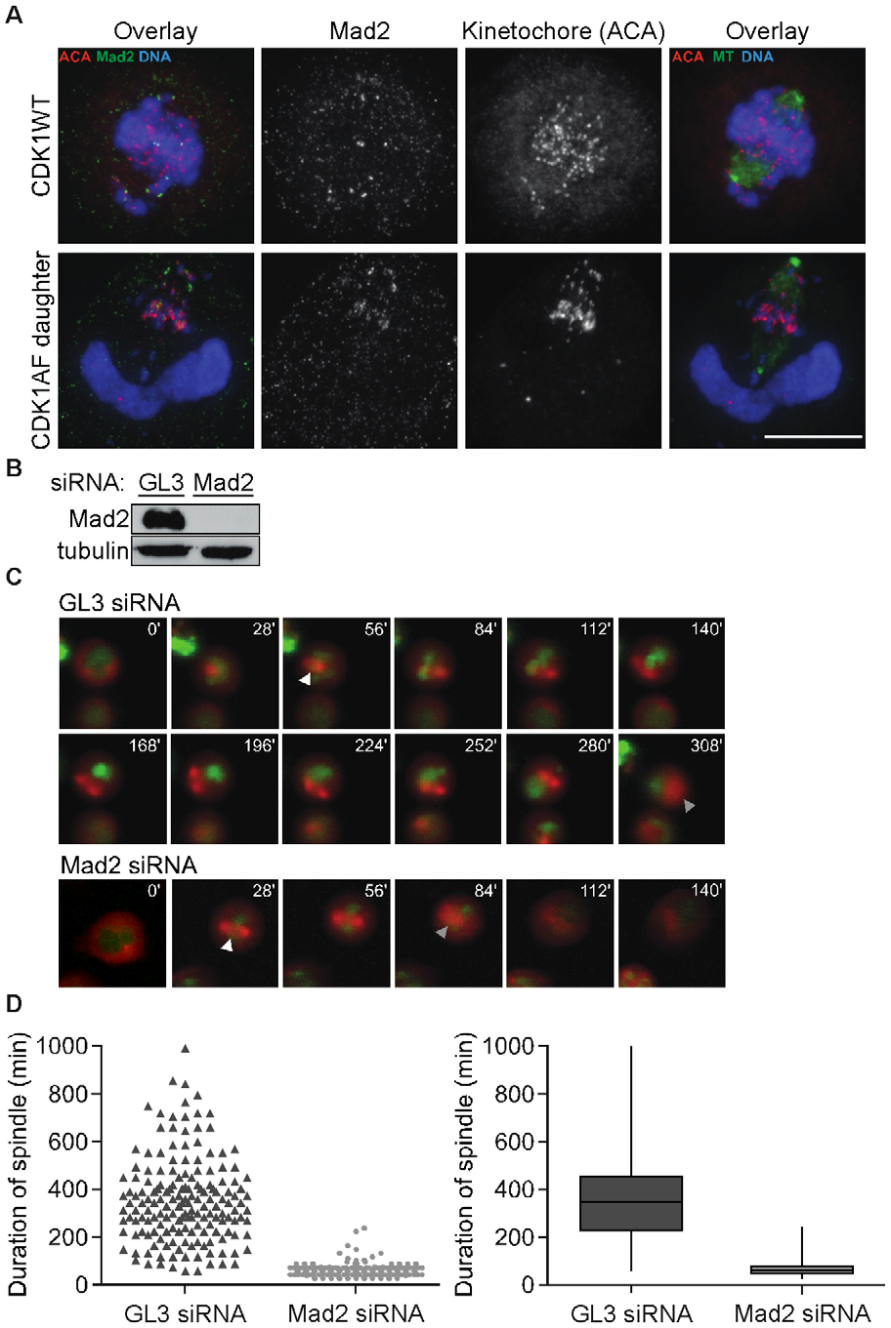
Mad2 stabilizes prematurely formed spindles in M-phase-like G1 cells. (A) Mad2 (green) with kinetochores (ACA; magenta) and DNA (blue) (left), or their overlay of MT (green) and DNA with kinetochores (ACA; magenta) (right) in a representative CDK1WT mitotic cell (top) and daughter produced by division of a CDK1AF-expresser (bottom). Scale bar = 10 µM. (B) Mad2 (top) and tubulin (bottom) immunoblots in a cell line stably expressing histone H2B-GFP (green) and mCherry-α-tubulin (red), following co-transfection with CFP-CDK1AF and Diced siRNA pools to firefly luciferase (GL3) or human Mad2 3′ UTR. (C) Montage of live-cell images from transfections described in (B) showing a representative GL3 d-siRNA-treated cell (top) and Mad2 d-siRNA-treated cell (bottom). White arrowheads indicate prematurely formed spindle; gray arrowheads indicate last image of apparent spindle structure. (D) Scatter plot of spindle duration times for CDK1AF/GL3-siRNA (dark gray) and CDK1AF/Mad2-siRNA (light gray) co-transfected cells (left) and box plot (right). Transfections and imaging were performed in triplicate (N = 3; n_GL3_ and n_Mad2_ = 167 representative cells). GL3-siRNA spindle duration: range, 60–990 min; first quartile, 225 min; median 345 min; third quartile, 450 min. Mad2-siRNA spindle duration: range, 30–240 min; first quartile, 45 min, median, 60 min; third quartile, 75 min.

Prior work described that oscillations of cyclin B1-YFP could occur in G1 daughters of CDK1AF-expressing cells, but it was unknown if these were enabled at least in part by the stabilization and activities of APC targets—including AurB—and undetermined if the SAC was active in these cells [8]. To address this, we probed CDK1WT-expressing mitotic cells and M-phase-like daughters of CDK1AF-expressing cells for Mad2, a key component of the SAC complex [35]. In prometaphase CDK1WT-expressing cells, kinetochores of unaligned chromosomes—mostly located at the spindle periphery—co-localize with Mad2 (Figure 4A, top). Surprisingly, despite incoherence in chromosome condensation and the resulting breakage of chromosome arms, the daughters of CDK1AF-expressing cells also presented Mad2 co-localization at some kinetochores (Figure 4A, bottom), implying possible SAC activity. We therefore hypothesized that the SAC plays a role in delaying this spindle disassembly, and tested if knocking down Mad2 in CDK1AF-expressing cells would relieve this phenotype – if the SAC were inactive, Mad2 removal would not accelerate spindle disassembly. GL3-d-siRNA-treated control cells were still conferred Mad2 protein (Figure 4B, “GL3”) and maintained their prematurely formed and highly stabilized spindles for extended periods, with a median duration of 345 min (Figure 4, C and D, “GL3”; Video S5). Mad2-ablated cells (Figure 4B, “Mad2”), however, disassembled their spindles much sooner, after a median period of 60 min (Figure 4C and D, “Mad2”; Video S6). These data show that the mitotic spindles formed prematurely in the M-phase-like daughters of CDK1AF-expressers generate a functional SAC, and this increased the duration of spindles by several-fold.

### SAC Inactivation Increases Cyclin B1 Oscillation Frequency in Prematurely Cycling G1 Cells

**Figure 5:**
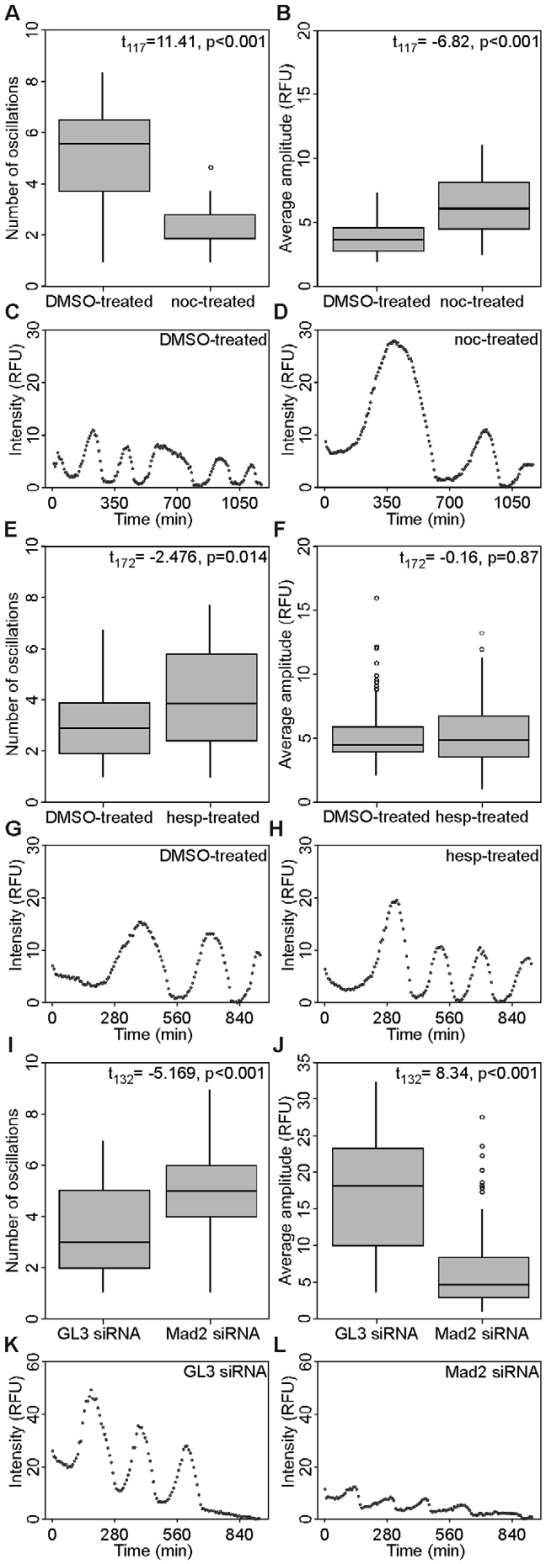
Mad2 knockdown in G1 cells with impaired APC-Cdh1 activity increases cyclin B1-YFP oscillation frequency. (A) Box plots of cyclin B1-YFP oscillation peak frequencies in DMSO- and nocodazole-treated cells, respectively. DMSO (oscillations per experiment (OPE)): range, 0.92–8.35; first quartile, 3.71; median, 5.56, third quartile, 6.49. Nocodazole (OPE): range, 0.92–3.71; first quartile, 1.85; median, 1.85; third quartile, 2.78. (B) Box plots of cyclin B1-YFP oscillation amplitudes in DMSO- and nocodazole-treated cells, respectively. DMSO (relative fluorescence units (RFU)): range, 1.9–7.3; first quartile, 2.7; median, 3.6, third quartile, 4.5. Nocodazole (RFU): range, 2.4–10.9; first quartile, 4.4; median, 6.0; third quartile, 8.1. (C, D) Representative cells oscillating at the median frequency in the DMSO and nocodazole treatments, respectively. (E) Box plots of cyclin B1-YFP oscillation peak frequencies in DMSO- and hesperadin-treated cells. DMSO (OPE): range, 0.96–6.74; first quartile, 1.92; median, 2.88, third quartile, 3.85. Hesperadin (OPE): range, 0.96–7.7; first quartile, 2.4; median, 3.85; third quartile, 5.77. (F) Box plots of cyclin B1-YFP oscillation amplitudes in DMSO- and hesperadin-treated cells, respectively. DMSO (RFU): range, 2.0–8.5; first quartile, 3.9; median, 4.4, third quartile, 5.8. Hesperadin (RFU): range, 1.0–11.2; first quartile, 3.5; median, 4.8; third quartile, 6.6. (G, H) Representative cells oscillating at median frequency in the DMSO and hesperadin treatments, respectively. (I) Box plots of cyclin B1-YFP oscillation peak frequencies in GL3- and Mad2 d-siRNA-treated cells. GL3 (OPE): range, 0.99–6.94; first quartile, 1.98; median, 2.97, third quartile, 4.96. Mad2 (OPE): range, 0.99–8.92; first quartile, 3.96; median, 4.96; third quartile, 5.95. (J) Box plots of cyclin B1-YFP oscillation amplitudes in GL3- and Mad2 d-siRNA-treated cells, respectively. GL3 (RFU): range, 3.5–32.4; first quartile, 9.9; median, 18.0, third quartile, 23.2. Mad2 (RFU): range, 0.9–14.9; first quartile, 2.9; median, 6.9; third quartile, 8.3. (K, L) Representative cells oscillating at median frequency in the GL3- and Mad2-d-siRNA treatments, respectively. In (A), (F), and (J), outliers that are at least 1.5 times the interquartile distance away from their respective quartiles are presented as circles.

CDK1AF expression in HeLa cells produces G1 daughters with improperly accumulated mitotic kinases and Cdc20, leading to activation of the SAC and highly stabilized bipolar spindles. Mad2 knockdown in these cells induced spindle disassembly nearly 5 hours earlier than control cells, suggesting that the SAC prolonged the period of APC-Cdc20 inactivity and increased the spindle duration in these G1 cells. We therefore treated CDK1AF-transfected HeLa cells either pharmacologically or by Mad2 knockdown to determine if SAC function—and therefore, APC-Cdc20 activity—could be altered, by measuring changes in cyclin B1-YFP oscillations. Substantially significant differences were observed between the cyclical behaviors of populations incubated with DMSO and nocodazole (t_117_ = 11.41, p<0.001). DMSO-treated cells exhibited a median frequency of 5.6 cycles in triplicate experiments, whereas nocodazole treatment slowed cyclin B1 oscillations by nearly 3-fold (1.85 cycles) (Figure 5A; representative oscillation in Figure 5C). Additionally, the relative YFP fluorescence of nocodazole-treated cells oscillated with amplitudes 61% greater than control cells (t_117_ = −6.82, p<0.001) (Figure 5B; representative oscillation in Figure 5D). This reduction in frequency and increase in amplitude by nocodazole supported the hypothesis that the SAC is functional and can be engaged in these newly divided cells, but still did not prevent cyclin B degradation. This phenomenon of cyclin degradation occurring with an active SAC has been described previously as “mitotic slippage” [36]. Hesperadin treatment—and its inhibition of AurB—had been shown earlier to override the SAC [34]. In our studies, daughters produced by CDK1AF-expressing cells treated with this drug exhibited a modest 34% increase in oscillation frequency over cells in DMSO—from 2.9 to 3.9 oscillations—per 16-h imaging experiment (t_172_ = −2.476, p = 0.014) (Figure 5E; representative oscillation in Figure 5G)—but did not significantly alter the amplitudes between the two (t_172_ = −0.16, p = 0.87) (Figure 5F; representative oscillation in Figure 5H).

We next tested whether Mad2 knockdown would change both the frequency and amplitude of cyclin B1-YFP oscillations. Daughters produced by division of GL3 siRNA/CDK1AF-transfected cells oscillated on average 3 times during the 16-h sample period, whereas cells knocked down for Mad2 nearly doubled that with an average of 5 oscillations (t_132_ = −5.169, p<0.001) (Figure 5I; representative oscillation shown in Figure 5K). Mad2 ablation also decreased the average amplitude of oscillations by 2.6-fold compared to the control (t_132_ = 8.34, p<0.001) (Figure 5J; representative oscillation shown in Figure 5L). Together, these data support the hypothesis that APC-Cdc20 activity can be influenced by factors that activate or inactivate the SAC in G1 cells that still possess M-phase-regulating proteins, here as a result of constitutively active CDK1 expression prior to cell division. By altering the oscillation frequencies and amplitudes of cyclin B1-YFP, we confirmed that premature spindles formed in these cells are capable of generating a SAC response.

### Expression of Non-Degradable Wee1 Reduces Premature Mitotic Onset in G1 Daughters Produced by Cells Expressing CDK1AF

Expression of a constitutively active mutant of CDK1 in HeLa cells permits cell division, but leads to premature mitotic spindle assembly in newly divided G1 cells. Surprisingly, these spindles were stabilized for several hours due to activation of the SAC. This revealed for the first time that APC activity sufficient for cell division but insufficient for complete proteolysis of mitotic substrates could contribute to bipolar spindle formation in G1 cells. This APC defect was initiated by CDK1AF expression, and it remained possible this Wee1-insensitive kinase became active prematurely to cause the observed mitotic behaviors. However, Wee1 is degraded in mitosis and absent during G1 phase [37], [38], [39], so it was plausible that the endogenous CDK1 would remain uninhibited—due to the absence of Wee1—and mediate this precocious cycling. We therefore analyzed whether or not Wee1 is present in mitotic cells, as well as the newly formed G1 cells that exhibit spindle formation soon after mitosis as a result of CDK1AF expression.

**Figure 6:**
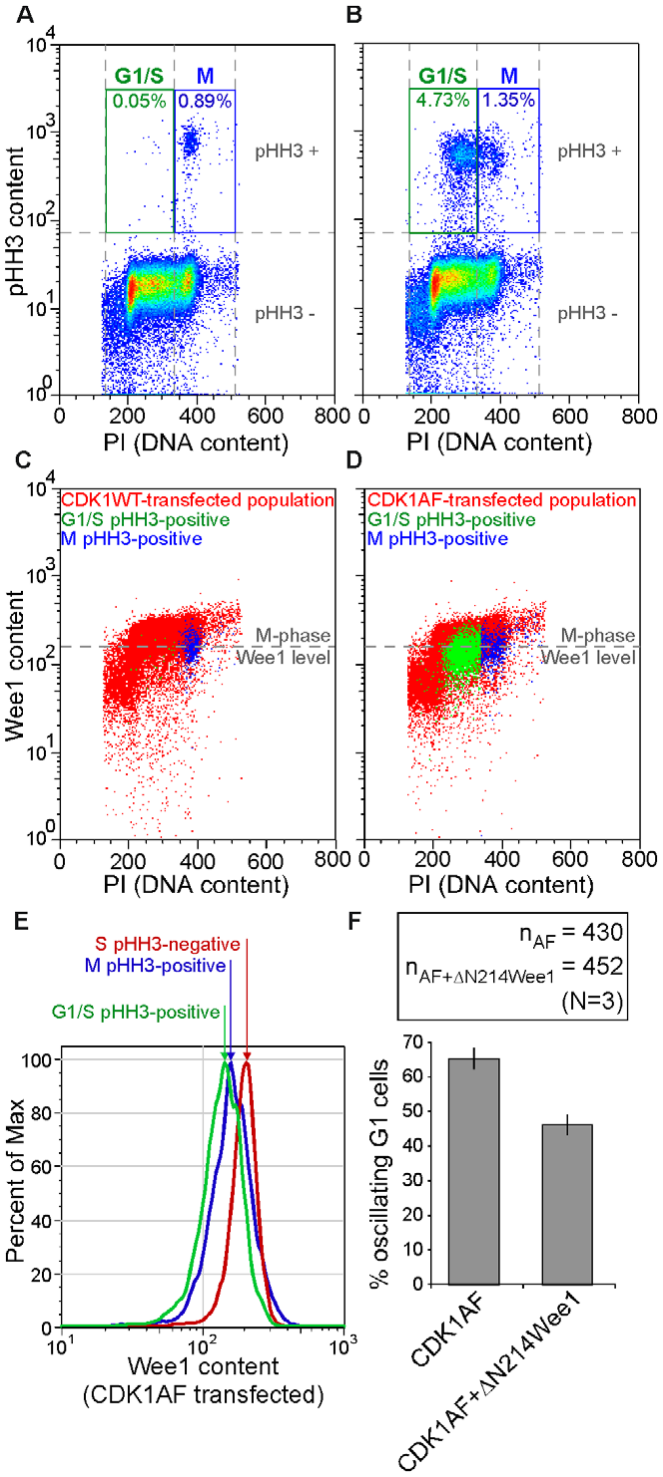
Premature M-phase initiation can be reduced in G1 cells with impaired APC-Cdh1 activation by enforced non-degradable Wee1 expression. (A) Fifty-thousand CDK1WT-transfected cells and (B) 50000 CDK1AF-transfected cells on a pseudo-color scatter plot of PI (abscissa) and pHH3 content (ordinate). High DNA and high pHH3 content (M phase) gates (blue “M”) and low DNA and high pHH3 (abnormal G1/S cells) gates (green “G1/S”) are shown with their respective frequencies. (C) Cells gated in (A) are overlaid on a pseudo-color scatter plot of PI (abscissa) and Wee1 content (ordinate) of CDK1WT-transfected cells. (D) Cells gated in (B) are overlaid on a pseudo-color scatter plot of PI (abscissa) and Wee1 content (ordinate) of CDK1AF-transfected cells. The M-phase Wee1 level (gray dashed line) is defined in both (C) and (D) by the horizontal axis centered on the Wee1-stained M-phase population that was gated in (A). (E) Histogram of relative Wee1 contents in CDK1AF-transfected cells in normal S phase (pHH3-negative; red), in normal M phase (pHH3-positive; blue), and in precocious M phase (G1-S DNA content, pHH3-positive; green) (F) Histogram of percent oscillating daughters generated in HeLa cells co-transfected with cyclin B1-YFP and CDK1AF alone, or together along with ΔN214Wee1.

CFP-CDK1WT- or CFP-CDK1AF-expressing cells were probed for pHH3, Wee1, and DNA content, and analyzed by flow cytometry. Each population was then gated for high pHH3 content (as would be expected in M) and low (G1/S) or high DNA content (M). Both treatments yielded about 1% M-phase cells (Figure 6A, B; blue “M” gate). CDK1AF-transfection produced nearly 5% with pHH3-positive-plus-G1/S DNA content, in contrast to 0.05% in the CDK1WT-transfected sample (Figure 6A, B; green “G1/S” gate). All cells from each respective treatment (all cells shown in Figs. 6A, B) were then plotted as a function of DNA (PI) and Wee1 content (Figure 6C, D; red), and the pHH3-positive/cell-cycle-gated subsets were then overlaid on these plots. G1/S pHH3-positive cells from the CDK1WT-transfected population were negligible (Figure 6C; green). M-phase pHH3-positive cells, however, were abundant and contained low Wee1 protein content in contrast to cells with high DNA content (Figure 6C; compare “blue” to “red” scatters) – the central axis of this population was defined as the “M-phase Wee1 level” (Figure 6C, D; gray hashed line). Not only did CDK1AF-transfected cells that were M-phase pHH3-positive follow suit (Figure 6D; blue), but the subset of cells that represented the improper cycling daughter cells—G1/S pHH3-positive—also contained a low abundance of Wee1 (Figure 6D, green). Plotting Wee1 contents of the CDK1AF-transfected cells (Figure 6D) as a histogram confirmed that peak Wee1 levels of the M-phase- and G1/S-pHH3-positive cells were nearly identical, with both lower than the Wee1 content of normal S-phase cells (Figure 6E). These data supported that the G1 cells undergoing precocious spindle formation contained negligible Wee1 kinase (i.e., were at or below the level found in normal mitotic cells), as also is the case in G1 phase during normal cell cycle progression. Thus, the normal degradation of Wee1 kinase during mitosis reinforces the need for proper APC function to mediate the destruction of mitotic regulators and fully restore cells to an interphase state.

Spindle formation coinciding with the absence of Wee1 kinase in G1 cells implied that endogenous CDK1 could be activated as cyclin B improperly re-accumulated, and that these phenotypes were not merely due to an inability to inhibit CDK1AF in G1 cells. We therefore hypothesized that enforcing the expression of a non-degradable version of Wee1 in CDK1AF-expressing cells would decrease the number of daughters that initiate these G1-phase mitotic events. Wee1 protein accumulates during S and G2 phase, and is degraded at M phase [38], [40]. Its N-terminus contains three phosphorylation sites (S53, S121, and S123) critical for its SCF-dependent ubiquitylation and proteolysis [38], [39]. Truncation of its N-terminal 214 residues lengthens the half-life of Wee1 protein but does not affect its kinase activity [41], [42], [43], so we engineered a non-degradable mCherry-fused deletion mutant of Wee1 lacking its N-terminal 214 amino acids (ΔN214Wee1). Expression tests in HeLa cells revealed a product of 78 kDa relative to the 98 kDa of the endogenous protein, plus some smaller bands as a result of mCherry being cleaved (Figure S4). HeLa cells were co-transfected with cyclin B1-YFP, CDK1AF, and with or without ΔN214Wee1, and live-cell imaging was performed 24 h later. Control (CDK1AF alone) and CDK1AF/ΔN214Wee1 co-expressing cells that prematurely accumulated cyclin B1-YFP following cell division were identified and scored for the onset of its oscillations – here, the onset of oscillations would reflect the activity of APC as a consequence of CDK1 activation. CDK1AF expression alone generated 65% cyclin B1-YFP oscillating daughter cells, whereas co-expression of ΔN214Wee1 reduced this frequency by nearly 20% (Figure 6F). In summary, expression of a constitutively active CDK1 in dividing HeLa cells can yield daughters with stabilized mitotic regulators during G1 phase, and along with the contribution of endogenous CDK1, induce premature mitotic behaviors that include bipolar spindle formation and SAC function.

### Cyclin B Overexpression and Proteasome Inhibition Does Not Induce Premature Mitotic Entry in Cdh1-deficient Cells

Division of HeLa cells expressing a constitutively active version of CDK1 accelerated the onset of S and M phases during their G1-phase period. Stabilization of some mitotic targets of APC-Cdh1 coincided with these phenotypes, and the improper presence and activities of these proteins (e.g. cyclin B and AurB) were necessary for these phenotypes. This raised the question of whether or not cells simply devoid of Cdh1 could also be induced to enter mitosis prematurely. Despite having early and prolonged S phases, previous studies involving Cdh1 knockdown did not report the onset of precocious M-phase phenotypes [5], [17], [18]. This suggested that cells with APC-Cdc20 alone could still behave quite normally, being insensitive to the presence of improperly stabilized mitotic targets of APC-Cdh1 during interphase. We therefore hypothesized that even further increasing the expression of cyclin B1 in Cdh1-depleted cells would force them into mitosis prematurely, as we observed in G1 daughters of CDK1AF-expressing cells.

**Figure 7:**
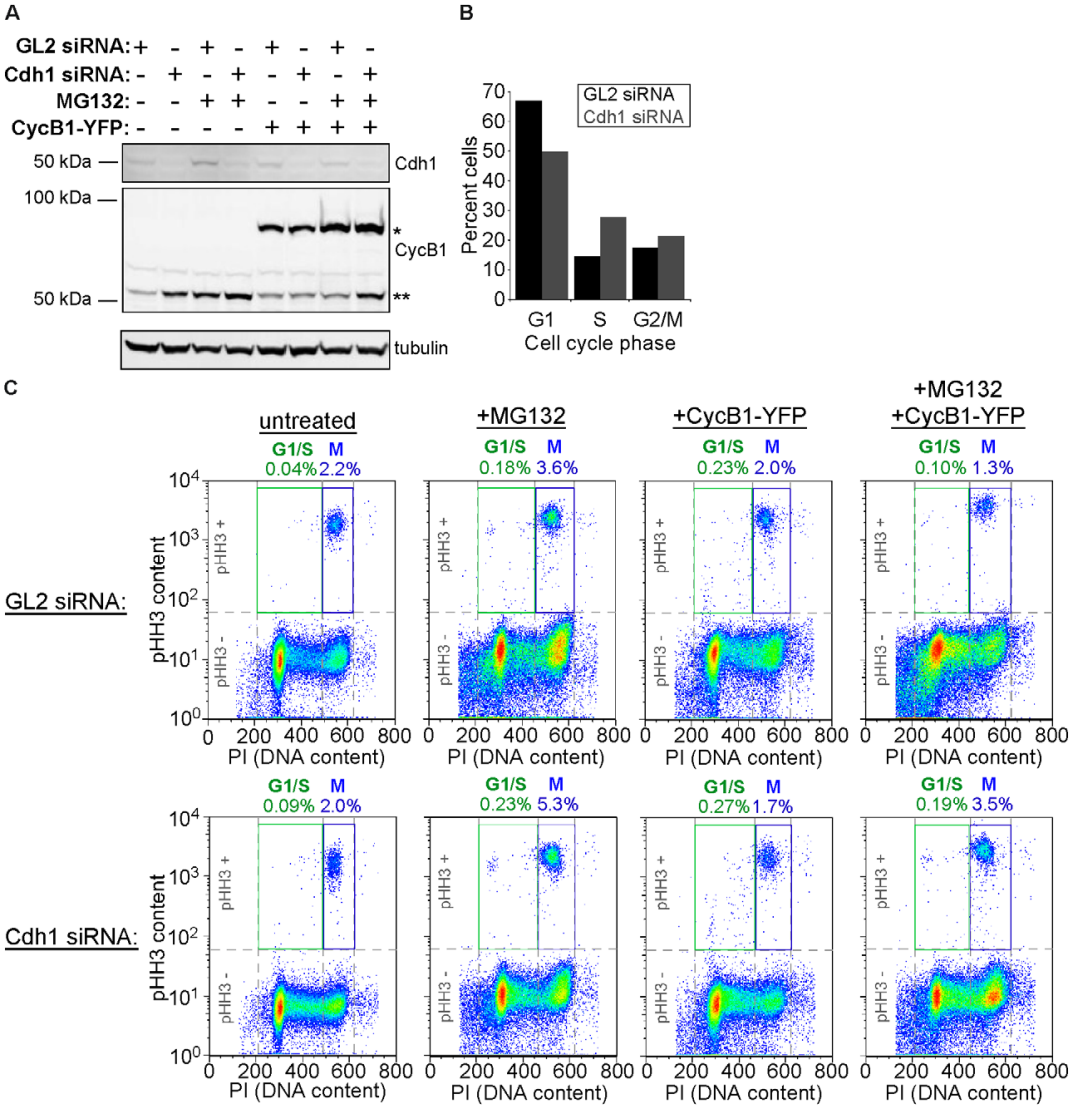
Cyclin B1 overexpression and proteasome inhibition fails to induce premature mitosis in Cdh1-ablated cells. (A) Western blots for Cdh1, cyclin B1 (CycB), and tubulin, of lysates from cells transfected with GL2 or Cdh1 siRNAs, followed by transfection with cyclin B1-YFP (CycB1-YFP) and/or treatment with proteasome inhibitor MG132. One asterisk indicates endogenous CycB, two asterisks indicates ectoptic CycB1-YFP. (B) Cell cycle distribution of GL2 and Cdh1 siRNA-transfected cells from (A) as determined by PI staining followed by flow cytometry. (C) Fifty thousand GL2-siRNA-transfected cells (top) and Cdh1-siRNA-transfected cells (bottom), either untreated or treated with MG132 and/or transfected with CycB1-YFP, on pseudo-color scatter plots of PI (abscissa) and pHH3 content (ordinate). High DNA and high pHH3 (M phase) gates (blue “M”) and low DNA and high pHH3 (abnormal G1/S cells) gates (green “G1/S”) are shown with their respective cell frequencies.

To test if Cdh1-ablated cells could be forced into a premature mitosis, HeLa cells were transfected with siRNAs directed towards Cdh1 and GL2 along with or without a construct encoding cyclin B1-YFP. Cells were then either left untreated or were incubated with MG132, to inhibit any potential proteasome activity and ensure maximum cyclin accumulation. Cdh1 ablation caused an increase in cyclin B1 relative to the GL2 control (Figure 7A), and this corresponded with an increase of S-phase cells (Figure 7B). However, both populations accumulated negligible low DNA-content/high pHH3-content cells, indicating that mitosis was still timed properly despite the lack of Cdh1 (Figure 7C, “untreated”). Surprisingly, this remained the case for Cdh1-ablated cells treated with MG132 (Figure 7C, “+MG132”), overexpressing cyclin B1 (Figure 7C, “+CycB1-YFP”), and under both conditions (Figure 7C, “+CycB1-YFP+MG132”; see Cdh1 and cyclin B1 levels in Figure 7A). Probing cyclin B1 in these populations revealed that its overexpression failed to induce premature M phase in cells in G1 and throughout S phase (Figure S5). This showed for the first time that cells ablated for Cdh1 not only manage to avoid a spontaneous accelerated mitotic entry, but also can resist significant increases in mitotic stimulus in the form of cyclin B1.

## Discussion

Mitotic progression and exit in eukaryotic cells requires the proteolysis of M-phase regulators. In somatic cells, the 26S proteasome-mediated degradation of proteins including cyclin B and securin requires their ubiquitylation by the earliest active form of the anaphase-promoting complex, APC-Cdc20. As CDK1 is inactivated, Cdc20 is exchanged for Cdh1, which then directs APC towards an additional set of targets, including Cdc20 and mitotic kinases AurA, AurB, and Plk1 [44], [45]. Positive feedback in CDK1 activation had been shown previously to be essential to ensure that APC activity is unimpeded at mitotic exit to permit sustained progressions through early embryonic and somatic cell cycles [7], [8]. Although HeLa cells expressing the positive-feedback-insensitive CDK1AF were found to divide successfully, their G1 daughters progressed into S phase prematurely, then exhibited phenotypes reminiscent of mitosis, including repeated NEB and NER events [8]. Using a fluorescent protein-tagged securin as an APC-Cdh1 biosensor in this latter study revealed that its activity was punctuated in the G1 daughters of CDK1AF-expressing cells. This led us to question whether hampering the sustained activity of APC-Cdh1 by CDK1AF expression might produce conditions more representative of phenomena that could arise *in vivo*, and potentiate behaviors that lead to dysregulated cell cycles and the onset of genomic abnormalities.

Here, we showed that CDK1AF expression permits HeLa cell division, but impeded APC-Cdh1 in completing the removal of some of its targets, contributing to a state permissive to premature mitotic spindle formation and SAC function in newly formed G1 cells. Numerous studies [5], [17], [18] and work in our laboratory (Figure 7) showed that elimination of Cdh1 by RNAi caused an accumulation of S-phase cells, but did not generate premature mitoses. In fact, Cdh1-ablated cells could not be forced to enter mitosis early, even with concurrent overexpression of cyclin B1 and treatment of these cells with proteasome inhibitor (Figs. 7 and S5). This surprising finding revealed that while Cdh1 removal accelerated the onset of S phase in HeLa cells, it did not increase their susceptibility for premature mitotic entry. One possible explanation for this could be that APC-Cdc20 activity can compensate for Cdh1 knockdown, resulting in a reduction in mitotic regulators that is sufficient to not permit premature mitotic entry. CDK1AF expression, however, could prevent the maximal activation of, and rapid switching between APC-Cdc20 and APC-Cdh1 during cell division, increasing the likelihood of an early mitotic onset. Additional studies will be performed to discern the mechanistic differences generated by either APC-Cdh1 knockdown or CDK1AF expression.

**Figure 8:**
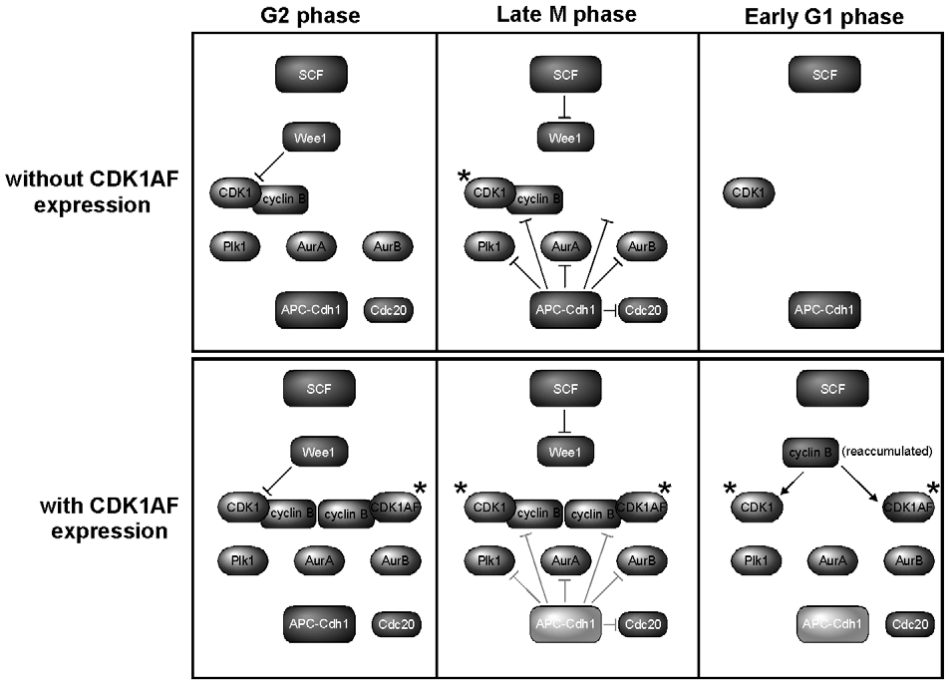
Overview of stabilized APC-Cdh1 targets and the absence of Wee1 in G1 daughters following division of cells with or without CDK1AF expression. Dark gray inhibitory symbols represent complete activation of APC-Cdh1; light gray inhibitory symbols (with CDK1AF expression) and light gray APC-Cdh1 represent a lack of sustained activation. Asterisks represent active CDK.

The fact that CDK1AF itself is incapable of being inhibited by Wee1/Myt1 kinases could mean that spindle formation and other M-phase-like phenotypes could somehow be attributed to the presence of this mutant CDK after mitotic exit. However, two lines of evidence from this work suggest that these precocious mitotic behaviors in G1 cells arise as a result of CDK1AF function during mitotic progression, and not simply due to its capability of being active in G1 phase. First, improper stabilization of the mitotic kinases and Cdc20 in G1 daughters support an APC-Cdh1 defect as a result of CDK1AF expression – this protein should have no direct involvement in preventing the proteolysis of M-phase regulators during cell division. Second, the absence of Wee1 in G1-phase HeLa cells—and the ability for non-degradable Wee1 to quell premature mitotic behaviors in daughters of CDK1AF-expressing cells—means that CDK1AF and the endogenous CDK1 are functionally equivalent at that point in the cell cycle. As schematized in Figure 8, cells with and without CDK1AF expression proteolyze Wee1, whereas the former also causes the improper stabilization of APC-Cdh1 substrates in G1 phase. Therefore, the endogenous CDK1 is active and capable of driving these behaviors along with the other residual mitotic regulators, no matter the contribution of CDK1AF during G1 phase in these precocious cycling cells.

One of the proteins left behind in CDK1AF-expressing daughter cells, AurB, controls MCAK during mitosis [46] and microtubule dynamics at the kinetochore [47], [48], and phosphorylation of Ser10 on HH3 during DNA condensation is attributable to this kinase [31]. As CDK1 activity rises and AurB is activated during the observed precocious mitoses, spindle formation occurs and is prolonged due to activation of the SAC (Figure 6). The finding that cyclin B-YFP levels oscillate in these cells and can be affected by nocodazole or inhibitor treatments demonstrates SAC function, but it is somehow less effective than in a normal mitotic cell. As shown in prior studies, cells arrested in prometaphase by the SAC (e.g. after their treatment with the spindle poison, nocodazole) are capable of slow cyclin degradation and eventual mitotic exit through “mitotic slippage” [36]. While the SAC is functional in G1 daughters yielded by division of CDK1AF-expressing cells, we propose that an exaggerated mitotic slippage occurs after they enter mitosis prematurely, and that this slippage may be more pronounced due to their containing reduced numbers of kinetochores and/or other SAC-regulatory components. Past studies demonstrated that mitosis with unreplicated genomes (MUG) and SAC activation can be induced in cells by chemically arresting them at the beginning of S phase for prolonged periods [49]. Our findings are novel in their showing that stabilized APC-Cdh1 targets in HeLa cells can not only increase the likelihood of cell cycle defects in newly produced daughters, but also provide a basis for functional mitotic spindles to form without completed DNA replication. We propose that these types of subtle change in APC-Cdh1 activity and subsequent effects on cell cycle progression could possibly increase the potential for aberrant genomic alterations to occur.

The overexpression of APC-Cdh1 substrates has been correlated with different types of malignant tumor, implying that Cdh1 plays a pivotal tumor-suppressing role [50], [51]. This underscores the need for maintaining proper dosage and activity of Cdh1 in the somatic cell cycle. Indeed, suppression of Cdh1 has been linked to many cancers [43], [52], and Cdh1 has been proposed to be a haploinsufficient tumor suppressor since *Cdh1* heterozygotic mice are more likely to develop spontaneous epithelial tumors [53]. Nevertheless, how defective APC-Cdh1 activation could lead to genomic instability and tumorigenesis on a cellular level remains elusive. Past studies involving Cdh1 knockdown described premature and prolonged S phase as the primary defect, without the onset of premature M-phase behaviors. While APC defects induced during mitotic exit by CDK1AF expression results in the production of inviable cells, it raises the possibility of serious implications as cells proliferate with incomplete destruction of mitotic targets. If cytokinesis occurs concurrently with a period of attenuated proteolysis, this could provide conditions permissive to premature cell cycle events and increase the potential for generating aneuploidy. Should premature mitotic entry occur in the presence of stalled replication forks and underreplicated DNA, the likelihood of segregating an unbalanced complement of chromosomes between daughters would be increased, and this loss of genomic integrity could be catastrophic. And as we observed as a result of CDK1AF expression, residual APC-Cdh1 targets remaining during G1 phase could indeed abrogate the proper timing of cell cycle transitions and cause conditions favorable for these types of defect. Tumorigenesis is often correlated with overexpression of cell cycle regulators, including the cyclins and M-phase kinases that are APC targets [51], [54]. Future studies into determining whether proteins such as these are truly being overexpressed in cancers—at the level of synthesis—or alternatively, are being incompletely degraded as cells exit mitosis, will shed new light onto how cells become permissive to neoplastic proliferation.

## Materials and Methods

### Cell Culture and Transfection

HeLa cells and stable HeLa cell lines expressing GFP-H2B and mCherry-α-tubulin as well as mCherry-α-tubulin alone (kindly provided by Claire Walczak) were cultured in DMEM (Mediatech, Manassas, VA) supplemented with 10% fetal bovine serum (Mediatech) and penicillin-streptomycin-glutamine (Mediatech, Manassas, VA.) in a humidified 37 deg C incubator with 5% CO_2_. For live-cell imaging, 1×10^4^ asynchronous cells were seeded into each well of a 96-well plate (BD Bioscience, San Jose, CA). Twenty-four hours later, cells were transfected with CFP-CDK1WT or CFP-CDK1AF (200 ng) with Lipofectamine LTX as per the manufacturer's instructions (Invitrogen, Carlsbad, CA), and were imaged live following an additional 24 h. For immunofluorescence studies, 2×10^5^ asynchronous cells were seeded into individual wells of a 6-well plate (BD Bioscience) each containing an acid-washed No. 12 circle coverslip (Fisher, Pittsburgh, PA). Cells were transfected twenty-four hours later with CFP-CDK1WT or CFP-CDK1AF (1 µg) using Lipofectamine LTX. For flow cytometry, 6×10^5^ cells were plated in 10 cm dishes, cells were left asynchronous or were synchronized by double-thymidine block and released, then transfected with 1.2 µg of CDKWT-CFP or CFP-CDK1AF plasmid using Genesilencer (Genlantis, San Diego, CA), according to the manufacturer's instructions. Cells were collected at 18 h and 30 h post-transfection and fixed in ice-cold 70% ethanol overnight at −20 deg C. For the non-degradable Wee1-plus-CDK1AF co-expression experiment, 2×10^5^ asynchronous HeLa cells were plated into 6-well plates and transfected 24 h later with CFP-CDK1AF, cyclin B1-YFP, and either WT- or ΔN214-Wee1-mCherry using Lipofectamine LTX. Cells were then replated into 96-well imaging plate 16 h after transfection, and 8 h later live-cell-imaging was performed for 17 h. For the Cdh1 knockdown, cyclin B overexpression/proteasome inhibition experiment, 2×10^5^ HeLa cells were plated into 6-well plates and blocked with 2.5 mM thymidine (Sigma) for 18 h. After being released, cells were transfected with siRNA oligos against luciferase GL2 or Cdh1 (Dharmacon, Lafayette, CO) using Oligofectamine (Invitrogen). Twenty-four hours later, cells were transfected with cyclin B1-YFP using Lipofectamine LTX, and then treated with 5 µM MG132 (Tocris Bioscience) for 16 h and collected and fixed in ice-cold 70% ethanol overnight at −20 deg C.

### Immunofluorescence

HeLa cells were fixed with pre-cooled methanol for 5 min at RT and rehydrated in PBS (137 mM NaCl, 2.7 mM KCl, 10 mM Na_2_HPO_4_, 2 mM KH_2_PO_4_, pH 7.4) for 2 min. Alternatively, cells were fixed with 3.7% formaldehyde in PBS for 15 min at RT, followed by permeabilization with PBS containing 0.3% Triton X-100 for 5 min at RT. Cells were stained with antibodies against α-tubulin DM1α (1∶1000) (Sigma-Aldrich, St. Louis, MO), nucleoporins mAb414 (1∶1000) (Abcam, Cambridge, MA), phospho-histone H3-pSer10 (pHH3) (1∶1000) (Sigma-Aldrich), Aurora A (1∶1000) (Abcam), Aurora B (1∶200) (Abcam), Plk1 (1∶200) (Abcam), cyclin B1 (Santa Cruz Biotechnology, Santa Cruz, CA), ACA (1∶100) (generated and kindly provided by Claire Walczak) and Mad2 (1∶200) (Santa Cruz Biotechnology) diluted in antibody diluting buffer (TBS, 2% bovine serum albumin, 0.1% Triton X-100, 0.1% sodium azide) for 1 h at RT. Cells were subsequently stained with 1∶200 dilutions of one or more of the following: donkey anti-rabbit Texas Red (Jackson ImmunoResearch, West Grove, PA), donkey anti-mouse Alex Fluor 488 (Invitrogen), and donkey anti-human Cy5 (Jackson ImmunoResearch) for 45 min at RT. Cells were mounted with Mowoil (Sigma-Aldrich) containing 1 µg/ml DAPI (Sigma-Aldrich). For the Cdh1 ablation, cyclin B overexpression, and proteasome inhibition experiment, 2×10^5^ HeLa cells were plated into 6- well plates and blocked with 2.5 mM thymidine (Sigma) for 18 h. After being released, cells were transfected with siRNA oligos against luciferase GL2 or Cdh1 (Dharmacon, Lafayette, CO) using Oligofectamine (Invitrogen). Twenty-four hours later, cells were transfected with cyclin B1-YFP using Lipofectamine LTX. Cells were then treated with 5 µM MG132 (Tocris Bioscience) for 16 h and collected and fixed in ice-cold 70% ethanol overnight at −20 deg C.

### Generation of ΔN214Wee1-mCherry Expression Construct

Non-degradable human Wee1 (ΔN214Wee1) was PCR engineered from full-length human Wee1 using the following primers: ACCGCTCGAGATGGATACAGAAAAATCAGGAAAAAGGGAATTTGATGT GCG conferring a Xho I site, and GCGCGGATCCGCAGCGTATATAGTAAGGCTGACA GAG conferring a BamH I site. The PCR product was then digested and cloned into a CMV-driven mammalian expression vector to produce an N-terminal fusion to mCherry fluorescent protein.

### Imaging

Fixed cells were imaged using a Leica DM5500B equipped with a 100× Apochromatic PLAN objective (NA 1.4) and a QImaging Retiga Exi FAST1384 CCD camera. The camera and filters were controlled via ImagePro (Bethesda, MD). Image stacks were collected at 0.5 µm steps throughout the entire cell volume and then deconvolved using Autodeblur (Autoquant Imaging, Bethesda, MD) for 10 iterations. For fluorescence intensity analysis, single-plane images were collected with a 40× PLAN APO objective (NA 1.0) at identical exposure times and fluorescence intensities were quantitated using ImageJ. High-throughput imaging was carried out with a BD Pathway 855 High-Content Bioimager system (BD Bioscience) using a 20× objective (NA 0.75) at a humidified 37 deg C with 5% CO_2_. Images were acquired using Attovision software (Beckton Dickinson) and analyzed using ImageJ software (NIH, Bethesda, MD).

### Cyclin B1 Oscillation Analysis

Given its cyclical nature, cyclin B1-YFP oscillations were analyzed using standard time series techniques. A linear trend was measured and removed from each cell leaving only the periodic pattern. A fast Fourier transform (FFT) combined with a modified Daniell smoother was used to calculate each cells periodogram. The peak frequency was recorded for each cell as cycles per sample then multiplied by the length of the time series with the resulting variable as number of cycles over time. Both the slope parameters, from the linear detrend and the peak frequencies were compared by within experiment t-tests. Each treatment (nocodazole, Hesperadin, and Mad2 siRNA) was compared to its own control with respect to time related decreases in intensity (linear trend) and frequency of fluorescence response (cyclical trend).

### Mad2 Knockdown

For knockdown of Mad2 in cells, a pool of Diced small-interfering RNAs (d-siRNAs) was generated against the 3′ UTR of Mad 2 as described [55]. Two-hundred-thousand Hela cells were plated per well in a 6-well plate, and were transfected 24 h later with 50 ng Mad2 d-siRNAs using Oligofectamine (Invitrogen) following the procedure recommended by the manufacturer. Primers for PCR amplification of the Mad2 transcriptional template from cDNAs generated from HeLa cells are as follows, with the addition of T7 promoter sequences to each primer: Mad2 forward primer: GCGTAATACGACTCACTAT AGGGGGATGACATGAGGAAAATAATGTA; Mad2 reverse primer: CCCTATAGTGA GTCGTATTACGCAGTCAAATAATTGGAAACTGA TTCA.

### Flow Cytometry

Cells frozen in 70% ethanol fixative were thawed, collected by centrifugation, washed with PBS, and incubated with 4.2 µg/ml anti-phospho-histone H3 antibody (clone 3H10; Millipore, Billerica, MA) and 2 µg/mL Cdc20 antibody (Santa Cruz Biotechnology) or 2 µg/ml Wee1 antibody (Santa Cruz Biotechnology) or 2 µg/mL cyclin B1 (Santa Cruz Biotechnology) in antibody diluting buffer (ADB; 1% bovine serum albumin and 0.1% Triton X-100 in PBS). After a 2 h RT incubation, cells were washed with PBS and stained with 4 µg/ml anti-rabbit (conjugated with Alexa Fluor 488) and 20 µg/ml anti-mouse Alexa Fluor 647-conjugated secondary antibodies (Invitrogen) in ADB for an additional 2 h. Cells were rinsed in ADB and resuspended in ADB with 50 µg/ml propidium iodide (Invitrogen) and 100 µg/ml RNase A. Quantitation of Cdc20,Wee1, cyclin B1 and pHH3 content was performed on a FACSCalibur and data was analyzed using FlowJo software.

### Immunoblotting

To determine the Mad2 knockdown efficiency, transfected cell lysates were prepared using M-PER mammalian protein extraction reagent (Pierce, Rockford, IL) 24 h after transfection and total protein concentration was determined by BCA assay (Pierce). Twenty micrograms of protein were separated by SDS-PAGE and transferred to polyvinylidene fluoride (PVDF). Blots were probed with antibody against Mad2 (1∶1000) (Santa Cruz Biotechnology) diluted in immunoblotting buffer (TBS-T containing 2% BSA and 0.1% sodium azide) overnight at 4 deg C. Membranes were then rinsed with TBS-T, probed with IRDye 800-conjugated anti-mouse secondary antibodies (1∶5000) for 1 h at RT, rinsed with TBS-T, and secondary antibodies were detected via the Odyssey infrared imaging system (LI-COR Biosciences, Lincoln, NE). For Wee1 and tubulin immunoblotting of recombinant Wee1-mCherry expression in HeLa cells, 30 µg of protein was loaded per lane, separated by 10% SDS-PAGE, transferred to PVDF, and probed with antibodies specific for Wee1 (1∶1000) and α-tubulin (1∶5000) diluted in immunoblotting buffer. This membrane was then stripped in 25 mM Glycine (pH 2.0) with 1% SDS and, and re-probed again with anti-mCherry antibody (BioVision, Mountain View, CA) (1∶5000), followed by probing and detection as described. For Cdh1 and cyclin B1 immunoblotting, 35 µg of protein was loaded per lane, separated by 10% SDS-PAGE, transferred to PVDF, and probed with antibodies against Cdh1 (1∶300) (Abcam), cyclin B1 (Santa Cruz Biotechnology) and tubulin (1∶5000).

## Supporting Information

Figure S1 **G1 daughters produced by CDK1AF-expressing cells possess broken chromosome arms surrounding and within their spindles.** Cells depicted in Figure 4, but staining singly for DNA (center images), (A) AurA, (B) AurB, or (C) Plk1 (right images), and overlays of both images (left images). Scale bars = 10 µM.
(TIF)

Figure S2 **Chromosome fragments with attached kinetochores are trapped within spindles of G1 daughters produced by division of CDK1AF-expressing HeLa cells.** DNA (blue), kinetochores (ACA; red), and the overlay of both of these in a daughter of a CDK1AF expresser. Scale bars = 10 µM.
(TIF)

Figure S3 **G1 daughters produced by division of CDK1AF-expressing HeLa cells form highly precocious and stabilized mitotic spindles.** Unsynchronized HeLa cells stably expressing histone H2B-GFP (green) and mCherry-α-tubulin (red) were transfected with CFP-CDK1WT or CFP-CDK1AF, then imaged live 24 h later (A and B). (A) Montage of CFP-CDK1WT-expressing cell undergoing normal mitotic progression. White arrowhead indicates mitotic cell. (B) Montage of CFP-CDK1AF-expressing G1 daughter cell undergoing premature and prolonged M-phase-like event. White arrowhead indicates premature M-phase-like G1 daughter. (C) Scatter plot of the spindle duration times for mitotic CDK1WT-expressing cells (light gray) and CDK1AF-expressing daughters (dark gray). (D) Box plot of cells shown in (C). Transfections and imaging were performed in triplicate (N = 3; n_CDKWT_ = 80 representative cells, n_CDK1AF_ = 80 representative cells). CDK1WT spindle duration: range, 22–66 min; first quartile, 26 min; median, 33 min; third quartile, 44 min. CDK1AF daughter spindle duration: range, 80–504 min; first quartile, 175.5 min, median, 252 min; third quartile, 324 min.
(TIF)

Figure S4 **Immunoblots of endogenous Wee1 and ΔN214Wee1 expression.** Thirty micrograms of total cellular lysate from untransfected cells (-) and ΔN214Wee1-transfected cells were probed with (A) Wee1 antibody, (B) mCherry antibody, and (C) α-tubulin antibody. mCherry truncations are indicated by brackets. Predicted MW of endogenous Wee1: 98 kDa; ΔN214Wee1-mCherry: 78 kDa; ΔN214Wee1 alone: 49 kDa.
(TIF)

Figure S5 **Phospho-histone H3 and DNA content of cyclin B1 overexpressing cells.** Fifty thousand GL2-siRNA-transfected cells (top) and 50000 Cdh1-siRNA-transfected cells (bottom), either untreated or treated with MG132 and/or transfected with CycB1-YFP. Pseudo-color scatter plots of PI (abscissa) and cyclin B1 content (ordinate) (top row of each siRNA treatment) were gated for high cyclin B1 levels (blue gate), and these cells (blue) were overlaid upon scatter plots of the total cell populations (red). Respective percentages of cells with high cyclin B1 content are shown.
(TIF)

Video S1 **Time-lapse video of CDK1AF- and PCNA-YFP co-expressing HeLa cell during and following mitosis to measure G1 duration.**
(MOV)

Video S2 **Time-lapse video of CDK1WT- and PCNA-YFP co-expressing HeLa cell during and following mitosis to measure G1 duration.**
(MOV)

Video S3 **Time-lapse video of a mitotic CDK1WT-expressing HeLa cell stably expressing histone GFP-histone H2B (green) and α-tubulin-mCherry (red).**
(MOV)

Video S4 **Time-lapse video of twin G1 daughter cells stably expressing α-tubulin-mCherry and GFP-histone H2B, produced by division of a CDK1AF-expressing cell.**
(MOV)

Video S5 **Time-lapse video of the cell depicted in** **Figure 4C** **(top) montage.**
(MOV)

Video S6 **Time-lapse video of the cell depicted in** **Figure 4C** **(bottom) montage.**
(MOV)
