# Supplementary figures and images for: Expression of Constitutively Active CDK1 Stabilizes APC-Cdh1 Substrates and Potentiates Premature Spindle Assembly and Checkpoint Function in G1 Cells
Source: PLoS One. 2012 Mar 29;7(3):e33835. doi: 10.1371/journal.pone.0033835 (PMC3315497; doi:10.1371/journal.pone.0033835)

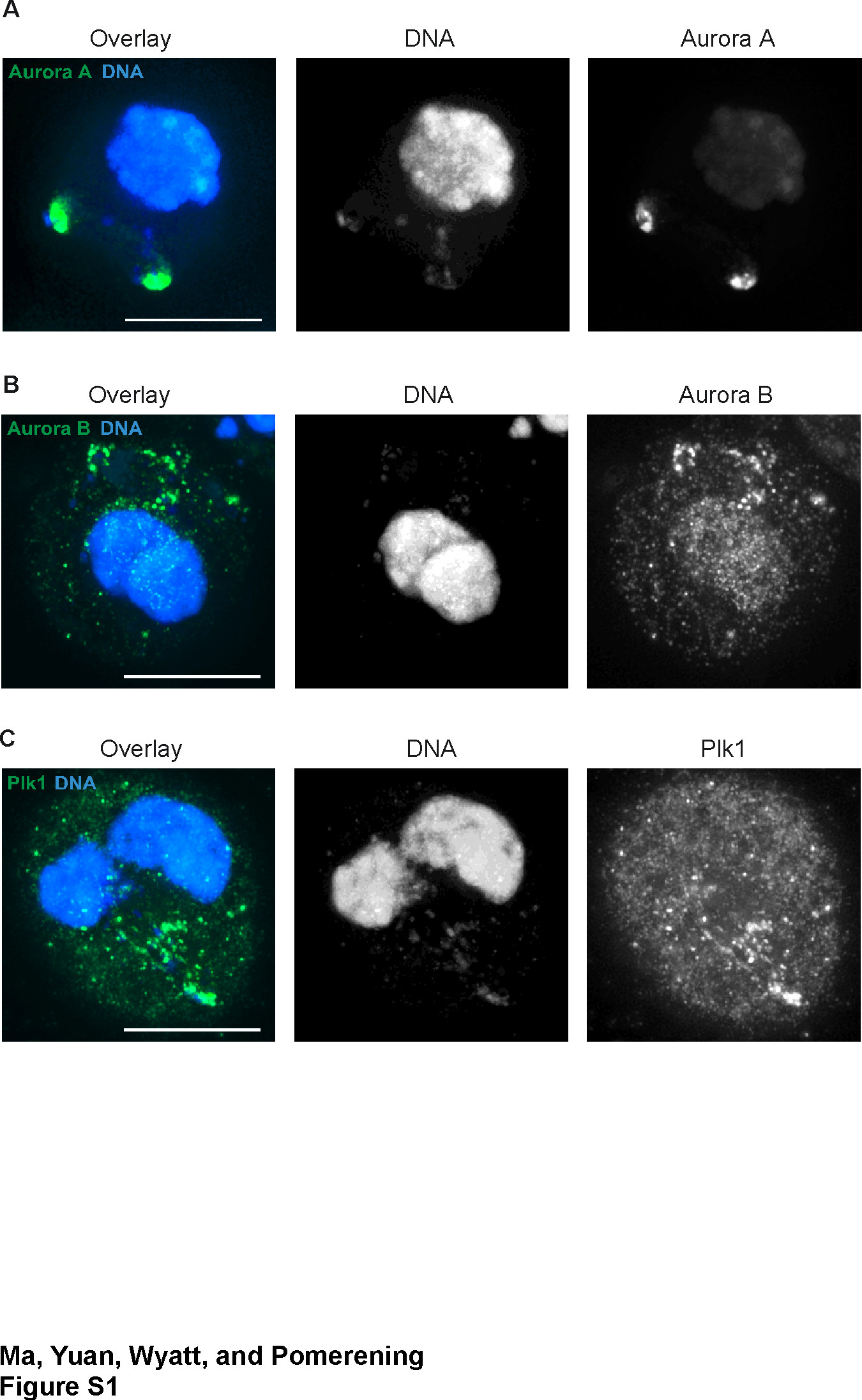

Supplement: Figure S1 — G1 daughters produced by CDK1AF-expressing cells possess broken chromosome arms surrounding and within their spindles. Cells depicted in Figure 4, but staining singly for DNA (center images), (A) AurA, (B) AurB, or (C) Plk1 (right images), and overlays of both images (left images). Scale bars = 10 µM. (TIF) [file pone.0033835.s001.tif]

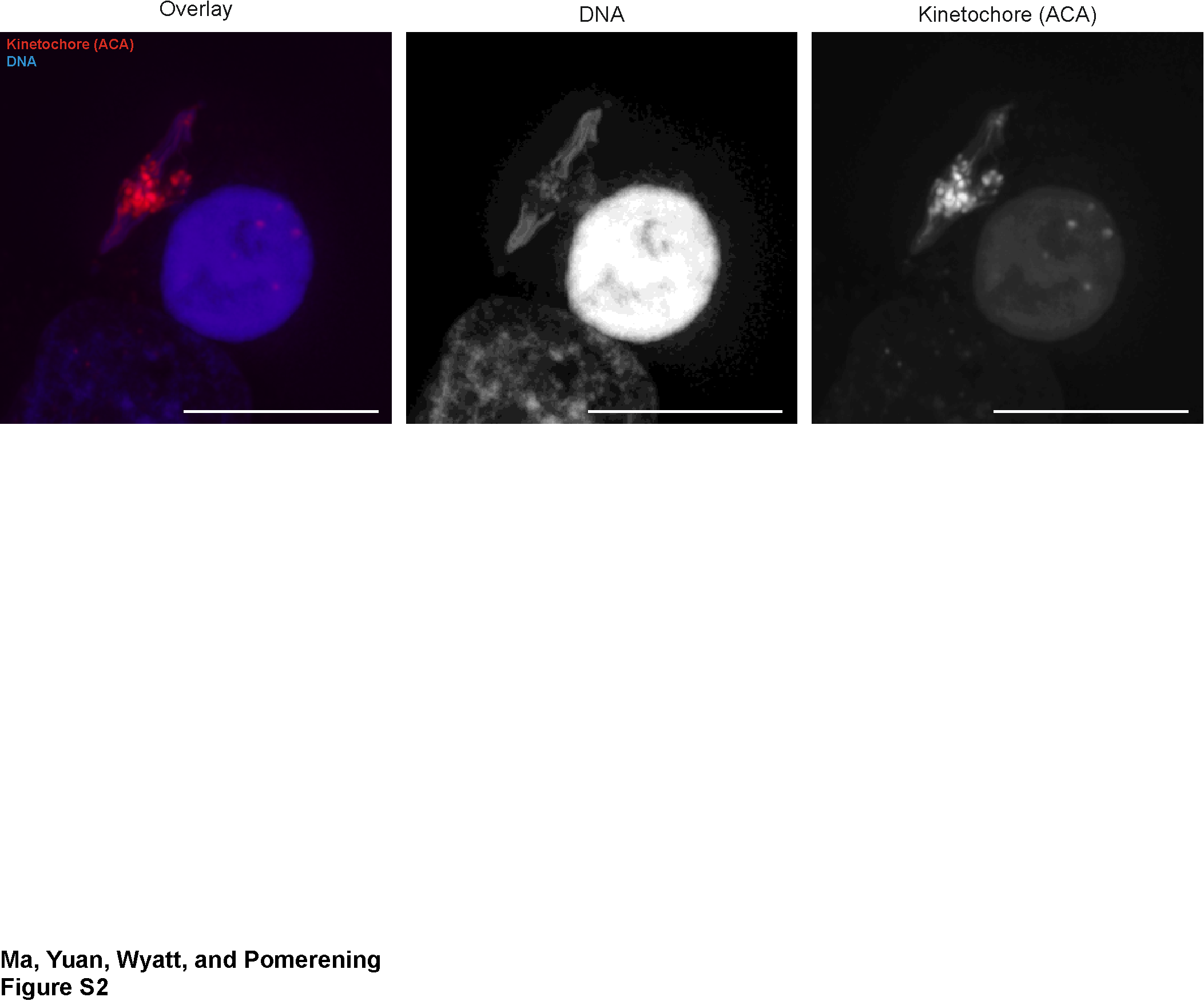

Supplement: Figure S2 — Chromosome fragments with attached kinetochores are trapped within spindles of G1 daughters produced by division of CDK1AF-expressing HeLa cells. DNA (blue), kinetochores (ACA; red), and the overlay of both of these in a daughter of a CDK1AF expresser. Scale bars = 10 µM. (TIF) [file pone.0033835.s002.tif]

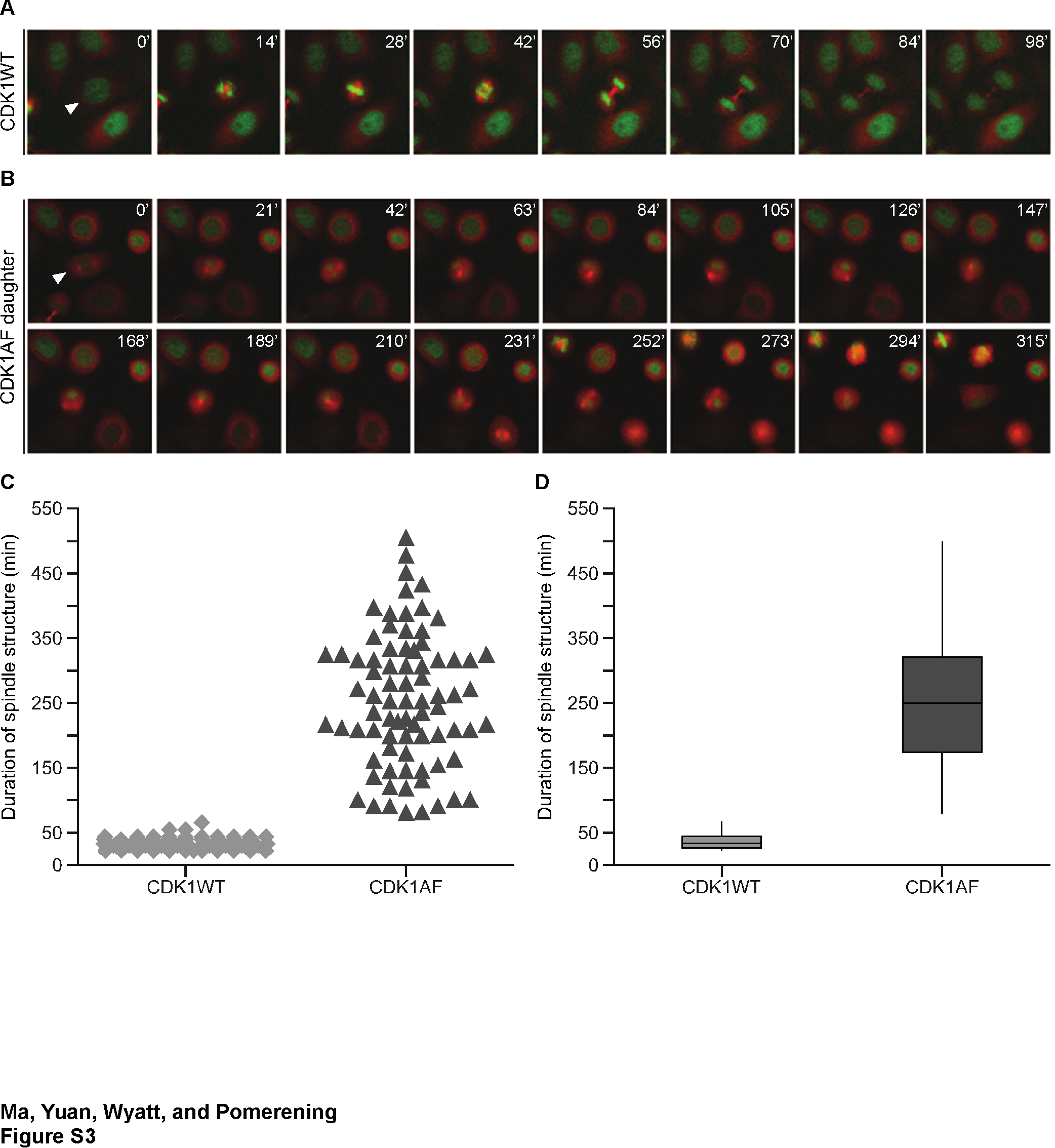

Supplement: Figure S3 — G1 daughters produced by division of CDK1AF-expressing HeLa cells form highly precocious and stabilized mitotic spindles. Unsynchronized HeLa cells stably expressing histone H2B-GFP (green) and mCherry-α-tubulin (red) were transfected with CFP-CDK1WT or CFP-CDK1AF, then imaged live 24 h later (A and B). (A) Montage of CFP-CDK1WT-expressing cell undergoing normal mitotic progression. White arrowhead indicates mitotic cell. (B) Montage of CFP-CDK1AF-expressing G1 daughter cell undergoing premature and prolonged M-phase-like event. White arrowhead indicates premature M-phase-like G1 daughter. (C) Scatter plot of the spindle duration times for mitotic CDK1WT-expressing cells (light gray) and CDK1AF-expressing daughters (dark gray). (D) Box plot of cells shown in (C). Transfections and imaging were performed in triplicate (N = 3; nCDKWT = 80 representative cells, nCDK1AF = 80 representative cells). CDK1WT spindle duration: range, 22–66 min; first quartile, 26 min; median, 33 min; third quartile, 44 min. CDK1AF daughter spindle duration: range, 80–504 min; first quartile, 175.5 min, median, 252 min; third quartile, 324 min. (TIF) [file pone.0033835.s003.tif]

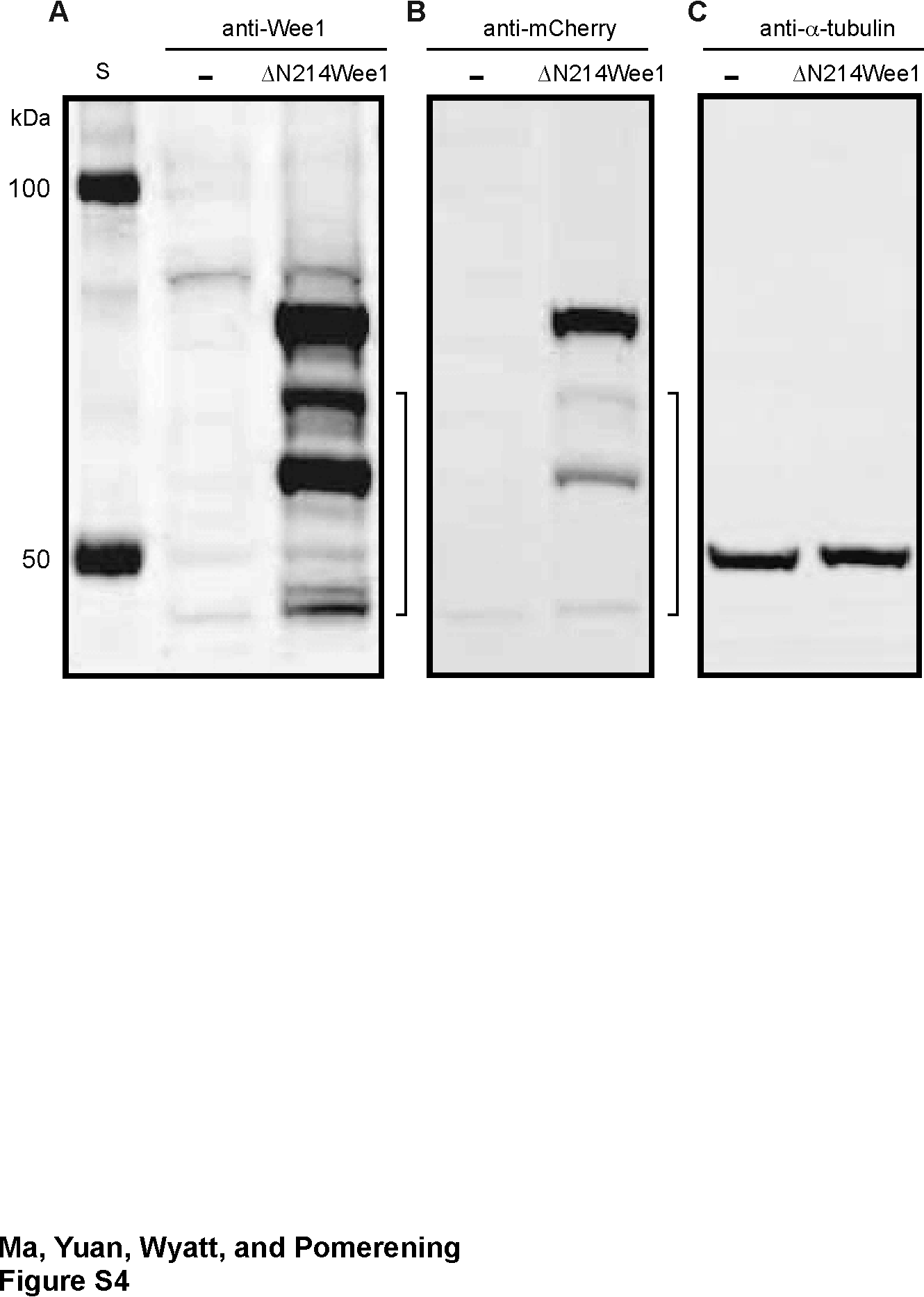

Supplement: Figure S4 — Immunoblots of endogenous Wee1 and ΔN214Wee1 expression. Thirty micrograms of total cellular lysate from untransfected cells (-) and ΔN214Wee1-transfected cells were probed with (A) Wee1 antibody, (B) mCherry antibody, and (C) α-tubulin antibody. mCherry truncations are indicated by brackets. Predicted MW of endogenous Wee1: 98 kDa; ΔN214Wee1-mCherry: 78 kDa; ΔN214Wee1 alone: 49 kDa. (TIF) [file pone.0033835.s004.tif]

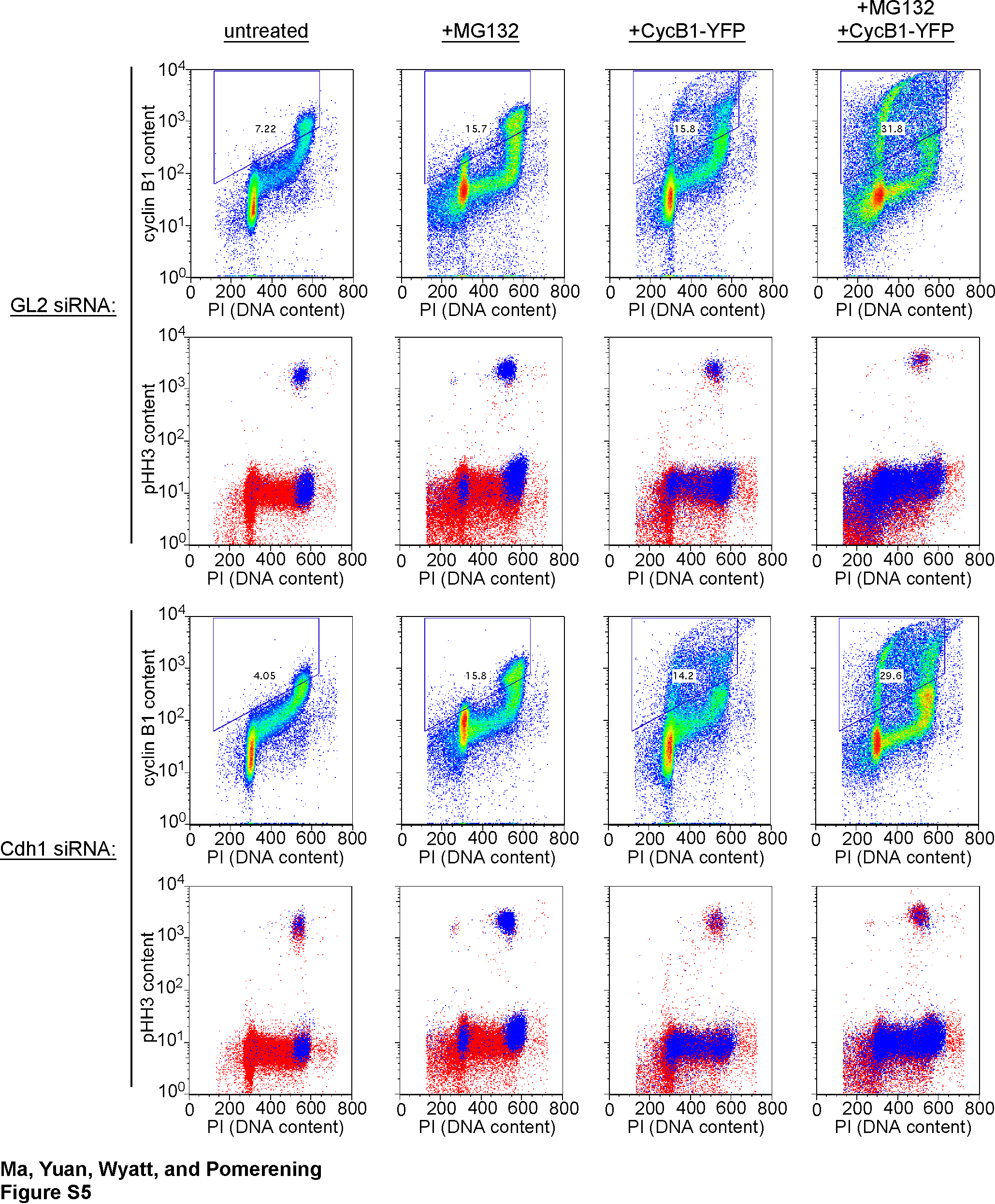

Supplement: Figure S5 — Phospho-histone H3 and DNA content of cyclin B1 overexpressing cells. Fifty thousand GL2-siRNA-transfected cells (top) and 50000 Cdh1-siRNA-transfected cells (bottom), either untreated or treated with MG132 and/or transfected with CycB1-YFP. Pseudo-color scatter plots of PI (abscissa) and cyclin B1 content (ordinate) (top row of each siRNA treatment) were gated for high cyclin B1 levels (blue gate), and these cells (blue) were overlaid upon scatter plots of the total cell populations (red). Respective percentages of cells with high cyclin B1 content are shown. (TIF) [file pone.0033835.s005.tif]
